# Health research capacity building of health workers in fragile and conflict-affected settings: a scoping review of challenges, strengths, and recommendations

**DOI:** 10.1186/s12961-021-00725-x

**Published:** 2021-05-22

**Authors:** Rania Mansour, Hady Naal, Tarek Kishawi, Nassim El Achi, Layal Hneiny, Shadi Saleh

**Affiliations:** 1 Global Health Institute, American University of Beirut, Beirut, 1107 2020 Lebanon; 2 St. George’s, University of London, London, UK; 3 Saab Medical Library, American University of Beirut, Beirut, Lebanon

**Keywords:** Health research, Capacity building, Fragile settings, Conflict-affected, Health workers

## Abstract

**Background:**

Fragile and conflict-affected settings (FCAS) have a strong need to improve the capacity of local health workers to conduct health research in order to improve health policy and health outcomes. Health research capacity building (HRCB) programmes are ideal to equip health workers with the needed skills and knowledge to design and lead health-related research initiatives. The study aimed to review the characteristics of HRCB studies in FCASs in order to identify their strengths and weaknesses, and to recommend future directions for the field.

**Methods:**

We conducted a scoping review and searched four databases for peer-reviewed articles that reported an HRCB initiative targeting health workers in a FCAS and published after 2010. Commentaries and editorials, cross-sectional studies, presentations, and interventions that did not have a capacity building component were excluded. Data on bibliographies of the studies and HRCB interventions and their outcomes were extracted. A descriptive approach was used to report the data, and a thematic approach was used to analyse the qualitative data.

**Results:**

Out of 8822 articles, a total of 20 were included based on the eligibility criteria. Most of the initiatives centred around topics of health research methodology (70%), targeted an individual-level capacity building angle (95%), and were delivered in university or hospital settings (75%). Ten themes were identified and grouped into three categories. Significant challenges revolved around the lack of local research culture, shortages in logistic capability, interpersonal difficulties, and limited assessment and evaluation of HRCB programmes. Strengths of HRCB interventions included being locally driven, incorporating interactive pedagogies, and promoting multidisciplinary and holistic training. Common recommendations covered by the studies included opportunities to improve the content, logistics, and overarching structural components of HRCB initiatives.

**Conclusion:**

Our findings have important implications on health research policy and related capacity building efforts. Importantly, FCASs should prioritize (1) funding HRCB efforts, (2) strengthening equitable international, regional, and national partnerships, (3) delivering locally led HRCB programmes, (4) ensuring long-term evaluations and implementing programmes at multiple levels of the healthcare system, and (5) adopting engaging and interactive approaches.

**Supplementary Information:**

The online version contains supplementary material available at 10.1186/s12961-021-00725-x.

## Introduction

Health research is rarely given the needed attention in low- and middle-income countries (LMICs), especially in fragile and conflict-affected settings (FCASs) [1–4]. Health research is pivotal in these settings given its potential to generate the necessary evidence to identify, address, and improve the well-being of a population [2, 5–7]. For instance, the knowledge produced from health research can inform the development and delivery of evidence-based health interventions, policies, and health systems tailored to the needs of a specific context or population [8, 9]. Despite that, FCASs lack adequate health research outputs and infrastructure due to multiple reasons such as the prioritization of immediate aid and relief efforts, military support, and implementation of peace-building initiatives, to name a few [10–15].

Although available funding to address health challenges relevant to FCASs along with the number of journals in this field is growing [16, 17], this has been largely driven by expertise and governance from high-income countries (HICs) to LMICs [18–21]. This is evidenced by the low authorship rates of LMIC authors within this field, as portrayed by a study in Lancet Global Health, which revealed that despite the fact that 92% of articles address interventions in LMICs, only 35% of authors are from LMICs [22]. The discordance between who is addressing and financing versus who is experiencing the specific challenges in FCASs has been associated with a neocolonialist model of global health [19]. Nevertheless, given numerous challenges faced in conflict settings, it is unsurprising that health research in FCASs is often funded and conducted by international institutions [2, 3, 23]. Their prominent role in humanitarian relief operations as well as their access to qualified research personnel abroad make them especially capable of conducting health research while operating in FCASs [12]. Yet the research initiatives funded and conducted by HIC entities are often temporary, unsustainable, lacking in local relevance, and often mirror the interests of HIC researchers [12–15]. For this reason, among others, it is crucial for FCASs to have the capability to produce their own contextualized and locally relevant health research outputs.

FCASs tend to lack qualified research staff and academic institutions, suffer from increasing attacks against healthcare institutions during times of armed conflict, face demanding health needs of populations living in chronic fragility and unstable sociopolitical circumstances, and operate under fragmented and overwhelmed healthcare systems [10, 11, 24]. This in turn makes it challenging for institutes in FCASs to strengthen research capacity, design and implement contextualized and sustainable solutions to local health problems, and focus on enhancing their research outputs.

Health research capacity building (HRCB) is a mechanism to simultaneously address the lack of health research and to strengthen the vulnerable healthcare systems in FCASs. It can be defined as a mechanism for “enhancing the abilities of individuals, organizations and systems to undertake and disseminate high quality research efficiently and effectively” [25, 26]. Accordingly, HRCB programmes have a strong potential to equip health workers in FCASs with essential tools and skills to design and conduct timely and contextually relevant health research projects. Health workers, as defined by WHO, are divided into two groups: health service providers, which are professionals who provide care such as physicians, nurses, dentists, therapists among others; and health management and support workers which are professionals not directly engaged in the provision of services and may include programme managers, policy-makers, and Ministry of Health staff among others [27]. Health workers are especially fit for HRCB programmes given that their role in the healthcare sector involves facing and tackling the local challenges of a fragmented health system [27]. They are thus uniquely positioned to define and address health research issues of importance and relevance to their population.

HRCB initiatives that aim to identify local issues and provide local solutions are likely to garner support from local policy-makers, programme managers, and funders, and may provide a better opportunity for the implementation and delivery of long-term and sustainable solutions [19]. Indeed, existing HRCB programmes in FCASs targeting health workers have revealed enhanced opportunities to define, develop, and tackle emerging health issues such as those resulting from conflicts, while also working towards achieving the sustainable development goals. Leading examples of such interventions include: field epidemiology and training programmes (FETP), a 2-year applied public health programme developed by the Centers for Disease Control (CDC) Epidemic Intelligence Services (EIS) and adopted globally, including in FCASs, by health ministries during disasters and humanitarian crises; in both situations FETPs have contributed to long-lasting results by training and working with local professionals to identify and tackle critical local problems [28]. FETPs have been implemented in over 80 locations following natural disasters to enhance local capacity in epidemiology methods and research, surveillance, and outbreak response [28]. Another project is the Research for Health in Conflict MENA (R4HC-MENA), a partnership between academic institutions in the United Kingdom and the Middle East and North Africa (MENA) [29]. R4HC-MENA aims to develop sustainable research capacity in the region as well as to improve knowledge and expertise in research methods to address major health challenges arising from conflict through the co-development and co-delivery of courses with faculty from the United Kingdom and MENA region.

Existing literature on HRCB has often focused on the practice and policy implications of HRCB and on exploring methods of translating research into policy and practice [30–34]. However, such studies have not focused on HRCB interventions conducted within FCASs, potentially because it is a relatively novel field, and thus there is a strong need for an overview of the state of this field in the past decade to help inform its future development. The aim of this scoping review is therefore to examine the current literature on HRCB in FCASs and to map such initiatives in order to support the identification of gaps and opportunities in HRCB across these settings. This will inform researchers, programme managers, policy-makers, and donors of past experiences, lessons learned, and potential opportunities for future work. Specifically, this review’s objectives are to: (1) identify characteristics of health research capacity building activities implemented across FCASs, (2) analyse their associated challenges and successes, and (3) recommend future directions for HRCB programmes in FCASs.

## Methods

### Design and search strategy

We conducted a scoping review to explore HRCB initiatives for health workers in FCAS. We followed the Preferred Reporting Items for Systematic Reviews and Meta-Analyses for Scoping Reviews (PRISMA-ScR) guidelines during the preparation of this review [35]. A scoping review was conducted as opposed to a systematic review, because the aim is to explore the type of available evidence on this topic and understand the extent of work within a field that is in its early development, rather than assess the data and quality of selected studies [36]. According to Arksey and O’Malley, scoping reviews are generally used to identify knowledge gaps, which aligns with our current aims.

We ran the same search strategy on the following academic electronic databases: Scopus, Embase, Cumulative Index to Nursing and Allied Health Literature (CINAHL), and Cochrane CENTRAL on 4 May 2020. We used the three concepts “Health Research”, “Health Workers”, and “capacity building”, under which we added all possible terms (see full search strategy in Additional file 1: Appendix 1). We also added a fourth condition to specify the selection of articles from FCASs, as informed by the World Bank specifications [37]. An example of all terms used under each concept in addition to their definitions is reported in Table 1 along with the full list of countries targeted.

**Table 1:** Search terms and definitions

Concept	Definition	Exemplar terms
Capacity building	The development of knowledge, skills, commitment, structures, systems, and leadership to enable effective health promotion ... [with] actions to improve health at three levels: the advancement of knowledge and skills among practitioners; the expansion of support and infrastructure for health promotion in organizations; and the development of cohesiveness and partnerships for health in communities [26]	Search capacity building[MeSh:noexp] OR healthcare literacy[tw] OR health literacy[tw] OR program literacy[tw] OR teaching[tw] OR teachings[tw] OR teachback[tw] OR teach-back[tw] OR course[tw] OR courses[tw] OR webinar[tw] OR webinars[tw] OR e-learning[tw] OR e-learning[tw] OR learning[tw] OR learnings[tw] OR online-learning[tw] OR education[tw] OR educational material*[tw] OR healthcare information[tw] OR health information[tw] OR health promotion[tw] OR healthcare promotion[tw] OR health programs[tw] OR health program[tw] OR health, etc.
Health research	Research that focuses on health-related topics	Search "Dual Use Research"[MeSh:noexp] OR "Research Subjects"[MeSh:noexp] OR "Genetic Research" [MeSh:noexp] OR Research[MeSh] OR "Research Support as Topic" [MeSh:noexp] OR "Ethics, Research" [MeSh:noexp] OR "Stem Cell Research" [MeSh:noexp] OR Research*[tw] OR medicine-investigation*[tw] OR ((Health*[tw] OR retrospective[tw] OR cohort[tw] OR prospective[tw]) AND (Volunteer*[tw] OR participant*[tw]))
Fragile and conflict-affected settings (FCASs)	According to the World Bank, these countries include: 1) High-intensity conflict countries: Afghanistan, Central African Republic, Libya, Somalia, South Sudan, Syrian Arab Republic, Yemen Republic; 2) Medium-intensity conflict countries: Burkina Faso, Burundi, Cameroon, Democratic Republic of Congo, Iraq, Mali, Niger, Nigeria, Sudan; and 3) High institutional and social fragility countries: Chad, Comoros, Congo Republic, Eritrea, Gambia, Guinea-Bissau, Haiti, Kosovo, Kiribati, Lebanon, Liberia, Marshall Islands, Micronesia, Myanmar, Papua New Guinea, Solomon Islands, Timor-Leste, Tuvalu, Venezuela, Zimbabwe, West Bank, and Gaza [37]	Search Afghanistan*[tw] OR Central-African-Republic[tw] OR Libya*[tw] OR Somalia*[tw] OR South-Sudan*[tw] OR Syria*[tw] OR syrie*[tw] OR Yemen*[tw] OR Burkina-Faso*[tw] OR Burundi*[tw] OR Cameroon*[tw] OR Congo*[tw] OR Iraq*[tw] OR irak*[tw] OR Mali*[tw] OR Niger*[tw] OR Nigeria*[tw] OR Sudan*[tw] OR Chad*[tw] OR Eritrea*[tw] OR Gambia*[tw] OR Guinea-Bissau*[tw] OR Haiti*[tw] OR Kosovo*[tw] OR Leban*[tw] OR liban*[tw] OR lubnan*[tw] OR lobnan*[tw] OR Liberia*[tw] OR Myanmar*[tw] OR (Papua adj New adj Guinea*)[tw], etc.
Health workers	According to WHO, health workers are all paid workers employed in organizations or institutions whose primary intent is to improve health and can be divided into two groups. The first group comprises the people who deliver services—whether personal or nonpersonal—who are called “health service providers”; the second covers people not engaged in the direct provision of services, under the term “health management and support workers”. [27]	Search ((Health Personnel[MeSh] OR Students, Health Occupations[MeSh] OR health workforce[MeSh:noexp]) OR (Health examiner*[tw] OR medical examiner*[tw] OR clinic examiner*[tw] OR clinical examiner*[tw] OR Health assistant*[tw] OR hospital assistant*[tw] OR medical assistant*[tw] OR clinic assistant*[tw] OR clinical assistant*[tw] OR Health administrator*[tw] OR hospital administrator*[tw] OR medical administrator*[tw] OR clinic administrator*[tw] OR clinical administrator*[tw] OR Health supervisor*[tw] OR hospital supervisor*[tw], etc.

### Eligibility criteria

All records included in this review are qualitative, quantitative, and mixed-methods studies that reported in English a capacity building initiative conducted in a FCAS after the year 2010, that targeted health workers, and that was related to a health research topic. Eligible studies included peer-reviewed articles examining interventions with a capacity building component and included the following study types: evaluation reports, randomized controlled trials, case studies, or project reports. We excluded all commentaries, editorials, letters to the editor, cross-sectional quantitative studies, reviews, abstracts proceedings, poster presentations, and all interventions that do not have a capacity building component such as those restricted to awareness sessions, webinars, and so on. We also excluded all studies that did not relate to health research topics, that did not focus on health workers, that were not conducted in a FCAS, and that were conducted before 2010.

### Screening and selection process

Multiple stages were undertaken in this review, starting with the search process, which was conducted by LH, a medical librarian. Records retrieved by this search were compiled in one Endnote library and were shared with two reviewers (RM and TK). The two reviewers then removed all duplicates in a two-step process: the first was conducted automatically through the Endnote software, and the second was conducted manually to make sure all remaining duplicates not detected by the software were identified and deleted. Next, RM and TK each independently screened all articles in two phases: the first included title and abstract screening, whereas the second included full-text screening. Upon completion of each phase, HN was assigned to adjudicate the selection process and resolve disagreements between both reviewers. Next, one author (RM) extracted the data into a pre-established Excel sheet which included variables classified into three sections: bibliography (name of first author, corresponding author institution and location, date of publication, study design, and funding organization), intervention (population addressed, sample size, duration of the initiative, setting and country where the study was conducted, topic and objective of the intervention, type of capacity building initiative, modality of delivery, and evaluation approach), and outcomes (reported challenges and limitations, strengths, opportunities, and recommendations).

### Analysis

We used a descriptive approach when reporting the data, and we followed a thematic approach for the analysis of qualitative data. We followed Thomas and Harden’s approach to thematic analysis of qualitative data in similar reviews but modified it based on the aims of this paper [38]. The first step involves coding the text, including line-by-line coding of the data, and the second step involves developing descriptive themes, which requires grouping codes based on similarities and differences. In the third step, we generated analytical categories, moving beyond the findings from the primary studies to generate additional concepts or understandings. We modified the third step given that our review did not aim to synthesize the findings into higher-order concepts; rather, our categories were chosen a priori based on the primary aims of this scoping review. One author (RM) conducted this process, and two authors (HN and TK) adjudicated the codes, themes, and categories based on discussions between the three authors.

## Results

### Selection process

**Fig. 1:**
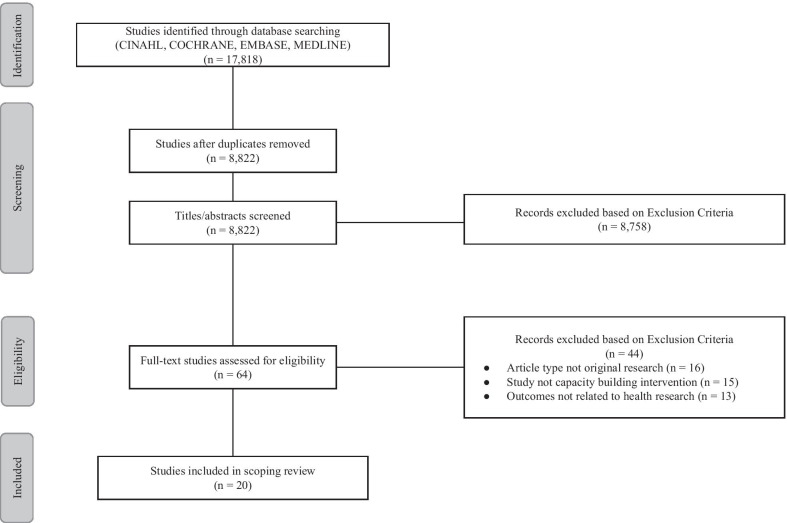
Preferred reporting items for systematic reviews and meta-analyses for scoping review (PRISMA-ScR) diagram applied during the scoping review, June 2020.

The initial search after removal of duplicates yielded 8829 articles, all of which were screened by title and abstract. Of those, 64 studies were selected for full-text review, and a total of 20 studies met our inclusion criteria (see Fig. 1).

### Characteristic of studies

**Fig. 2:**
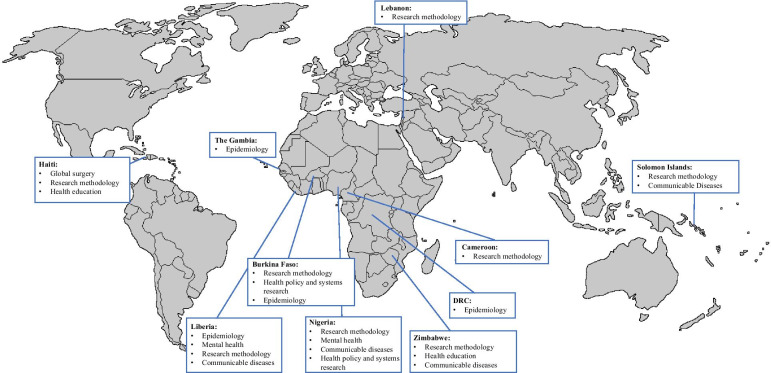
Visual representation of HRCB in FCASs

Tables 2 and 3 highlight characteristics of included studies. Included articles, as represented in Fig. 2, reflected an HRCB initiative conducted in Nigeria (*n* = 6) [39–44], Haiti (*n* = 4) [45–48], Zimbabwe (*n* = 4) [40, 49–51], Liberia (*n* = 3) [42, 52, 53], Burkina Faso (*n* = 2) [54, 55], Solomon Islands (*n* = 2) [56, 57], Dominican Republic of Congo (*n* = 1) [52], Cameroon (*n* = 1) [54], Gambia (*n* = 1) [52], and Lebanon (*n* = 1) [58], most of which were published after 2015 (85%). Those initiatives centred around topics such as general health research methodology (70%), communicable diseases (30%), global surgery (10%), health education (10%), health policy and systems research (10%), mental health (10%), and epidemiology (5%). Included articles were mixed-methods studies (60%), project reports (20%), qualitative studies (15%), or quantitative studies (5%). The reported initiatives delivered their capacity building programmes to academics (70%), health service providers (55%), health management and support staff (35%), and community members who were present as a subgroup in some programmes (15%). Noticeably, HRCB initiatives targeting health care workers and academics took place in all regions, whereas HRCB initiatives delivered to health management and support staff were only based in Africa. Additionally, only three of the studies reported the genders of their participants.

**Table 2:** Characteristics of included studies (*N* = 20)

Study characteristic	*N* (%)
*Region of corresponding author affiliation*	
Global north	15 (75%)
Global south	5 (25%)
*Region of first author affiliation*	
Global north	12 (60%)
Global south	8 (40%)
*Funding status*	
Government agency	11 (55%)
International organization	6 (30%)
University	3 (15%)
Not specified	2 (10%)
*Study design*	
Mixed methods	12 (60%)
Project report	4 (20%)
Qualitative	3 (15%)
Quantitative	1 (5%)
*Country*	
Nigeria	6 (30%)
Haiti	4 (20%)
Zimbabwe	4 (20%)
Liberia	3 (15%)
Burkina Faso	2 (10%)
Solomon Islands	2 (10%)
Cameroon	1 (5%)
Gambia	1 (5%)
The Dominican Republic of Congo	1 (5%)
Lebanon	1 (5%)
*Health topic*	
Health research methods	14 (70%)
Communicable diseases	6 (30%)
Global surgery	2 (10%)
Health education	2 (10%)
Health policy and systems research	2 (10%)
Mental health	2 (10%)
Epidemiology	1 (5%)
*Type of capacity building*	
Workshop	13 (65%)
Mentorship	5 (25%)
Fellowship	2 (10%)
Course	1 (5%)
Teaching rounds	1 (5%)
Training Programme	1 (5%)
Residency	1 (5%)
*Target population*	
Academics	14 (70%)
Health service providers	11 (55%)
Health management and support staff	7 (35%)
Community members (subgroup)	3 (15%)
*Level of implementation*	
Individual	19 (95%)
Organizational	4 (20%)
System	3 (15%)
*Setting*	
Mixed	10 (50%)
University	2 (10%)
Hospital	3 (15%)
Not specified	5 (25%)
*Mode of delivery*	
In person	13 (65%)
Blended	7 (35%)
Online	0
*Pedagogy*	
Interactive	11 (55%)
Practicum-based	9 (45%)
Theory	7 (35%)
*Evaluation time point*	
Pre/post/during intervention	3 (15%)
Short-term post intervention (<1 year)	15 (75%)
Long-term post intervention (≥ 1 year)	4 (20%)
Not specified	2 (10%)

**Table 3:** Bibliography of academic articles addressing HRCB of frontline health professionals across FCASs

Reference	Corresponding author; collaboration direction	Study design	Topic	Objective of HRCB intervention	Type of intervention	Target population (sample size)	Challenges and limitations	Strengths	Recommendations
*Africa*									
Mbuagbaw, 2013 [54]	North; South	Mixed methods	RM	To build clinical trials skills in Cameroon	Workshop	Academics; HMSS (not specified)	Language barriers	Context-specific design, practical exercises, interactive learning	Contextually relevant material, practical pedagogy
Olaleye, 2014 [39]	South; North-South	Process report	RM	To enhance institutional research culture as well as increase the knowledge and skills of resident doctors, graduate students, and faculty	Workshop	HSPs; academics (not specified)	Human resources, attitude	Local implementation	N/A
Dagenais, 2015 [55]	North; North-South	Mixed methods	RM; HPSR	To implement a knowledge-brokering (KB) project to encourage research use in a West African context	Training programme	HSPs; academics (28)	Local research context, sustainability, human resources, language barriers	Local implementation, practical exercises, interactive learning	Evaluation approach, preparation via needs assessment, contextually relevant material
Owiredu, 2017 [40]	North; North-South	Mixed methods	RM	To improve access, quality, and uptake of PMTCT services, by enhancing service delivery in health facilities through support of implementation research projects	Workshops; group training; staff mentorship	HSPs; HMSS; academics (3400)	Local research context, funding, attitude	Local collaborations, local implementation, comprehensive research training	Evaluation approach, equity in partnership involvement
Mc Kenzie Andre, 2017 [52]	North; North-South	Process report	Epi	To strengthen the capacity of countries to more rapidly detect, respond to, and contain public health emergencies at their source.	Workshops	HMSS (1354)	Sustainability, human resources, funding	Local implementation, multidisciplinary population	Training of trainers, mentorship
Aagaard, 2018 [49]	North; North-South	Mixed methods	HE	To enhance the educational capacity of UZCHS by developing skills in curriculum development, programme evaluation, and educational leadership for faculty	Workshop; mentorship	Academics (41)	Human resources, funding, duration, technology	Practical exercises, interactive learning, mentorship component, sustainability approach	Funding, duration
Ibrahim, 2019 [41]	North; North-South	Process report	RM	To advance the scholarship of nursing practice and improve patient care outcomes.	Grant scholarship with research mentorship	HSPs (1)	Partnerships, human resources, funding, duration, language barriers	Based on needs-assessment, cost effectiveness	Communication, contextually relevant material, mentorship
Zhou, 2019 [50]	South; South-North	Process report	RM; CD	To prepare junior academics for local and global research / to build individual and institutional research capacity in Zimbabwe	Fellowship	Academics (5)	N/A	Interactive learning	Funding, communication, resources, equity in partnership involvement, multidisciplinary systems approach, mentorship
Gureje, 2019 [42]	South; South	Mixed methods	MH; RM; CD;	To create an infrastructure to develop mental health research capacity in Sub-Saharan Africa and to advance global MH science by conducting innovative public health-relevant research in the region.	Fellowship; Workshops; with mentorship	Academics (35)	Partnerships, sustainability	Based on needs-assessment	Multidisciplinary systems approach, contextually relevant material, practical pedagogy
Mayor, 2019 [53]	North; North-South	Mixed methods	RM; CD	To build and strengthen health research capacities at the St. Joseph’s Catholic Hospital in Monrovia.	Workshops; e-learning mentorship programme	HSPs; HMSS; academics; community members (21)	Partnerships, funding, duration, technical resources, technology, attitude, learning barriers/AD, evaluation approach, evaluation tools, participant engagement	Local collaborations, practical exercises, multidisciplinary population, sustainability approach	Funding, evaluation approach, equity in partnership involvement
Evans-Lacko, 2019 [43]	North; North-South	Mixed methods	MH; RM	To develop evidence and capacity for mental health system strengthening in Ethiopia, India, Nepal, Nigeria, South Africa and Uganda.	Short courses	HSPs; academics; HMSS (293)	Partnerships, learning barriers/AD, evaluation tools	Context-specific design, interactive learning, multidisciplinary population	Training of trainers, evaluation approach
Jack, 2020 [51]	North; North-South	Mixed methods	RM	To develop and train trainers from three African countries to conduct a systematic review workshop at their home universities as part of a broader mental health research capacity building project.	Workshop	Academics; HMSS (14)	Technical resources, technology, learning barriers/AD, evaluation approach, evaluation tools	Context-specific design, cost effectiveness	Training of trainers, resources, evaluation approach, equity in partnership involvement
Onwujekwe, 2020 [44]	South; South	Qualitative	HPSR; CD	To determine the needs of producers and users of evidence in priority setting for HPSR in the control of endemic diseases; facilitate planning and implementing research and research uptake activities for the control of NTDs and malaria in their respective states	Workshop	Academics; HMSS (118)	Sustainability, funding, duration, evaluation approach, evaluation tools	Context-specific design, based on needs assessment, comprehensive research training	Equity in partnership involvement
*Caribbean*									
Swain, 2015 [45]	North; North-South	Mixed methods	GS	To improve resident training in academic global surgery. Secondary: To strengthen longitudinal partnerships with institutions in low-resource settings.	Residency	HSPs (3)	Partnerships, local research context, human resources, technology, attitude, evaluation approach	Local collaborations, mentorship component	Training of trainers, equity in partnership involvement
Minn, 2015 [46]	North; North-South	Qualitative	RM	To introduce participants to qualitative methods, to present examples of qualitative research that had been conducted in Haiti	Workshop	HSPs (not specified)	Partnerships, duration, attitude	Context-specific design, local collaborations	Duration, equity in partnership involvement
Elharram, 2017 [47]	North; North-South	Mixed Methods	RM; HE	To develop medical knowledge and skills via anatomy dissections, surgical simulations, clinical pathology shadowing, and interactive sessions in research methodology and medical education.	Workshops	Academics (24)	Duration	Practical exercises, interactive learning, mentorship component, mutually beneficial	Preparation via needs assessment
Kaseje, 2018 [48]	North; North-South	Mixed Methods	GS	To establish a paediatric surgical rotation. Secondary: To dedicate one day each week to didactic and research activities	Teaching rounds	HSPs (9)	Local research context, human resources, technical resources	Context-specific design, sustainability approach	N/A
*Pacific Islands*									
Redman-Maclaren, 2012 [57]	North; North-South	Qualitative	RM; CD	To implement a research capacity-strengthening workshop addressing topics in research methodology, with teaching strategies that included planning, conducting, and reporting of pilot studies on tuberculosis (TB), HIV, and intestinal parasitic worms amongst researchers and chiefs in the Solomon Islands and researchers in Australia	Workshop	HSPs; community members (48)	Local research context, funding, technical resources, language barriers, learning barriers/AD, evaluation approach	Local collaborations, local implementation, practical exercises, comprehensive research training, multidisciplinary population, mutually beneficial, cost effectiveness	Evaluation approach, preparation via needs assessment
Maclaren, 2015 [56]	North; North-South	Mixed methods	CD	To strengthen research capacity in a hospital and community in the Solomon Islands using a “learn-by-doing” process	Workshop	HSPs; community members; academics (8)	Local research context, sustainability, human resources, language barriers	Context-specific design, local collaborations, practical exercises	Evaluation approach
*Middle East*									
Dagher, 2016 [58]	South; South	Quantitative	RM	To systematically and reliably provide research experience to undergraduate students interested in entering the field of medicine	Volunteer programme	Academics (104)	Human resources, attitude, participant engagement	Local collaborations, mentorship component, comprehensive research training, mutually beneficial	Training of trainers, duration

*RM* research methodology, *CD* communicable diseases, *GS* global surgery, *Epi* epidemiology, *HE* health education, *HPSR* health policy and systems research, *MH* mental health, *PMTCT* prevention of mother-to-child transmission, *UZCHS* University of Zimbabwe College of Health Sciences, *NTDs* neglected tropical diseases, *HSPs* health service providers, *HMSS* health management and support staff, *AD* academic difficulties

Included studies primarily aimed to enhance the skills and knowledge of participants in health research methods, including theoretical and practical applications of qualitative and quantitative research, data collection, proposal development, clinical research, among others. As such, almost all of the studies targeted an individual-level capacity building angle (95%), and a smaller number addressed an organizational-level (20%) or system-level (15%) angle. The initiatives were mainly delivered in university or hospital settings (75%), in face-to-face format (65%), with less than half having an online aspect to them (30%), and with a minority reporting a long-term evaluation approach (20%).

With regards to publication characteristics, our results show that 75% of corresponding authors were affiliated with an institution from a country in the Global North, most commonly the United States and Canada. Similarly, 60% of first authors were affiliated with the Global North. Most studies were based on north-south collaborations, with locally led efforts in the Global South being minimal (25%). Almost half of the studies were published in journals with impact factors ranging from 0 to 2 (*n* = 12), and the rest in higher-impact journals between 2 and 8.

Of the 20 studies reviewed, 18 indicated their funding sources. Eleven studies (55%) reported funding from national government agencies, six studies (30%) reported funding from international organizations, and three (15%) studies reported funding from universities.

### Qualitative analysis

We centred our qualitative analysis around three major categories in accordance with the primary aims of the study. These categories include (1) challenges to implementing HRCB interventions, (2) strengths of the HRCB interventions, and (3) recommendations and opportunities for improvement. In each of these categories, we reported associated themes emerging from the analysis, along with codes and exemplar quotes (see Additional file 2: Appendix 2 for a full description). A summary of results from our thematic analysis is outlined in Table 4. This thematic synthesis approach was applied to all qualitative text labelled as “findings” or “results” within the studies analysed for this scoping review. Although one study was quantitative in nature, Thomas and Harden [38] explain that the qualitative component of a study includes all of the text labelled as “results” or “findings”. Hence, in order to ensure comprehensive analysis of the findings from all studies, this approach was implemented across all 20 studies

**Table 4:** Summary of thematic analysis

*Challenges to implementing HRCB interventions*	
Structural/systemic challenges	Underdeveloped research culture influenced the prioritization of HRCB programmes, the development of locally led national and regional partnerships, and the lack of sustainability of initiatives
Logistical challenges	Organization and execution of HRCB programmes was hindered due to a shortage of both technical and human resources as well as funding, lack of adequate time to conduct the programme, and issues of technological literacy
Personnel challenges	Miscommunication due to language barriers along with varying levels of acceptability and support towards HRCB by institutional leadership contributed to the delay in implementation of the programmes
Assessment and evaluation concerns	Gaps in collecting data from participants and stakeholders, including lack of appropriate tools, inadequate long-term assessment mechanisms, as well as low rate of participant engagement, made it difficult to accurately assess both proximal and distal outcomes of the HRCB programme
*Strengths of HRCB interventions*	
Locally driven	Initiatives developed by local collaborations, designed to meet local needs, and informed by local needs assessments were successful in ensuring that the HRCB interventions were beneficial to the population
Pedagogical considerations	HRCB programmes with interactive and practical pedagogical approaches were perceived as more engaging and beneficial to participants, particularly when they allowed participants to contribute to ongoing local projects
Holistic CB Intervention	Capacity building programmes that promoted inclusion of a multidisciplinary population and involved learning about all stages of research development, coordination, and delivery were reported as more acceptable, feasible, and sustainable
*Recommendations and opportunities for improvement*	
Logistics of HRCB development and delivery	There is a need for FCASs to prioritize local funding for HRCB initiatives, equip programmes with adequate resources to carry out the programme, ensure they are of a longer duration, and train trainers on understanding the needs and cultural aspects of the local context
Structural components of HRCB interventions	To overcome systematic issues, recommendations include conducting a needs assessment to subsequently tailor the HRCB programme, preparing a thorough evaluation approach, involving various stakeholders and disciplines, and ensuring equity in partnership involvement
Content of HRCB interventions	It is suggested that programmes be designed using contextually relevant material and delivered using engaging and practical approaches with hands-on experiences to facilitate active learning

#### Challenges

Four main themes emerged from the analysis of the challenges category, and these include (1) structural and systemic challenges, (2) logistical challenges, (3) personnel limitations, and (4) assessment and evaluation concerns.

##### Systemic challenges

Under the systemic challenges theme, our analysis revealed that included studies commonly reported problems associated with the local research context [40, 45, 48, 55–57]. The research culture in many FCASs is still underdeveloped, and health workers may not prioritize or give much importance to such activities as opposed to managing health projects or providing actual clinical services. In addition, health workers were often reported to be overwhelmed with other responsibilities such that they perceived health research as being additional and unnecessary work duties. Furthermore, the development and maintenance of regional and local partnerships was a common challenge, seeing that most studies were driven by north-south partnerships that lacked nationally led initiatives [41–43, 45, 46, 53]. This is problematic because such initiatives lacked adequate contextualization to address locally relevant health issues. Finally, this was also related to another systemic challenge associated with the sustainability of these programmes given that with the absence of locally driven governance and leadership, such programmes had very little chances of surviving and imparting long-term impact [42, 44, 52, 55, 56].

##### Logistical challenges

Logistical challenges included difficulties pertaining to the organization and execution of HRCB initiatives. Studies indicated that maintaining a consistent stream of participants was difficult. Many studies reported that registrants dropped out potentially because HRCB initiatives distracted them from original duties, while other studies revealed that there was a small number of staff and projects in FCASs to engage local participants, potentially due to staff turnover and the lack of a financial incentive to contribute to HRCB interventions [39, 41, 45, 48, 49, 52, 55, 56, 58]. Securing and maintaining local funding for HRCB initiatives was also considered a logistical challenge across FCASs. Studies reported that HRCB programmes were largely driven by funding from the Global North given the limited resources within FCASs, yet following implementation of HRCB, this scarcity in local funding led to limitations in follow-up and long-term support [40, 41, 44, 49, 52, 53, 57]. In addition, time allocated to conduct HRCB intervention was often less than adequate [41, 44, 46, 47, 49, 51, 53]; one particular study highlighted the risk of having week- or month-long programmes fall under “helicopter ethnography” and “voluntourism” [46], where HIC researchers engage in rapid, temporary, and often self-serving activities in LMICs without planning for or building long-term relationships in LMICs. Additionally, the use of technology such as tablets to expand access to HRCB initiatives was problematic without adequate training [45, 49, 51, 53]. This is because some trainees lacked sufficient computer literacy skills and thus found it difficult to adopt e-learning platforms. Insufficient technical resources, including intermittent electricity, internet, and printing services, were reported to exacerbate the difficulties of adopting technological interventions [48, 51, 53, 57]. It was also related to hindering collaboration with researchers internationally, such as when writing manuscripts.

##### Personnel challenges

The theme of personnel challenges highlighted problems relating to the individual participants of HRCB interventions. Studies revealed that local researchers and personnel in institutional leadership roles displayed different levels of acceptability towards the HRCB interventions [39, 40, 45, 46, 53, 58]. The lack of support towards HRCB programmes was reported to delay implementation of the programme as well as hinder/discourage learning by participants. Additionally, language barriers were a common challenge reported throughout the studies, given that the language used to deliver the HRCB intervention was at times not the first language of participants [41, 54–57]. This was reported to result not only in miscommunication between partner institutions, but it also led to misunderstandings of programme material among learners. A few studies also highlighted that the academic difficulty of material presented to participants was a learning barrier, particularly when the knowledge presented in the HRCB intervention was beyond the educational level of learners [43, 51, 53, 57].

##### Evaluation challenges

Our analysis also revealed that studies reported challenges concerning the assessment and evaluation of the HRCB interventions. Studies indicated that the low rate of participant engagement when tasked with evaluating the HRCB initiative limited the collection of adequate data regarding the success of HRCB interventions [53, 58]. Furthermore, studies reported concerns regarding the evaluation approach and evaluation tools used to assess the HRCB programmes [44, 45, 51, 53, 57]. In particular, a common gap was not collecting data from all members affected by the HRCB intervention, including community members, institutional leadership, and health workers not directly involved in the HRCB programme but whose work may be impacted by it. The studies discussed that this gap prevented researchers from fully determining the impact of the HRCB intervention on the broader organizations, community, and system levels over the long term. Additionally, studies reported the difficulty in assessing the practical and behavioural impact of HRCB interventions due to inadequate evaluation tools to assess such distal outcomes [43, 44, 51, 53]. In particular, studies highlighted the inadequacy of pre-/post-training tests and self-reported questionnaires at capturing the impact of the HRCB intervention on knowledge gained as some of the skills acquired cannot be quantified via such tools.

#### Strengths

Three main themes relating to the strengths of HRCB initiatives were highlighted in the selected studies, and they centred around them being (1) locally driven, (2) considerate of engaging pedagogies, and (3) holistic.

##### Locally driven initiatives

Locally driven initiatives demonstrated significant strengths, in that they were designed to meet specific needs relevant to the context in which they were implemented, as informed by local needs assessments [41–44, 46, 48, 51, 54, 56]. These initiatives were also driven by local collaborations and were implemented by local actors [40, 45, 46, 53, 56–58]. Having them driven and implemented by local actors allowed for knowledge sharing between partner institutions prior to the delivery of the HRCB initiatives and subsequent utilization of local examples and issues of interest throughout the HRCB material provided to learners [39, 40, 52, 55, 57]. Such context-specific design and implementation of HRCB activities prevented a neocolonialist approach to HRCB and ensured that the HRCB interventions were indeed beneficial and relevant to the FCAS population.

##### Interactive pedagogies

Another important strength was noted among initiatives that had special considerations for the pedagogy through which the material was delivered to participants. Common strengths were reported for initiatives that used interactive approaches that had a practical component, and those that emphasized matching participants with mentors [43, 49, 50, 54, 55, 57]. For example, studies that encouraged practical research tasks during the HRCB intervention reported that the task had benefited local projects being conducted outside of the HRCB intervention [47, 53, 56]. Additionally, including a mentorship component was reported to offer not only research guidance during and after the HRCB intervention, but also career and professional advice, particularly to novice researchers [45, 47, 49, 58].

##### Holistic initiatives

Studies that implemented a holistic capacity building intervention reported strengths related to providing comprehensive research training to a multidisciplinary population in a sustainable method. Being involved in all the stages of research was identified as important by participants, particularly among early-career researchers, as it provided them an opportunity to learn how to coordinate, conduct, and communicate their own research [40, 44, 57, 58]. Additionally, training cohorts that included participants from various health and professional sectors, including veterinary, laboratory, and community health workers for example, promoted further collaborations on local projects [43, 52, 53, 57]. Studies also reported an advantage among HRCB interventions that were mutually beneficial to both the local participants from FCASs as well as the partner institution, namely that they promoted a decolonizing framework to north-south partnerships [47, 57, 58]. Finally, HRCB initiatives implemented in a cost-effective manner and with a preplanned sustainability approach also demonstrated significant strengths [41, 48, 49, 51, 53, 57]. Notably, such interventions were reported as more acceptable, feasible, long-lasting, and empowering of the local community.

#### Opportunities and recommendations

Recommendations and opportunities reported throughout the included studies centred around three main themes, namely (1) logistics of HRCB development and delivery, (2) structural components of HRCB interventions, and (3) content of HRCB interventions.

##### Logistic recommendations

Under the logistics theme, studies highlighted several areas to be considered in future interventions. There is a strong need for FCASs to prioritize allocation of local funding for HRCB to reduce dependency on foreign donors [49, 50, 53], to equip programmes with increased resources [50, 51], and to improve the planning and implementation of such interventions on different levels. For example, studies commonly recommended that future interventions make sure to design longer-lasting programmes that consider the long duration typically required from participants to develop and disseminate research findings [46, 49, 58]. This also includes longer time spans that allow participants to engage and maintain communication with their mentors for continuous support [41, 50]. Finally, several studies recommended that future initiatives pay particular attention to adequately training their trainers on cultural awareness and diversity, teaching skills, and on understanding the needs of the target groups and local context [43, 45, 51, 52, 58].

##### Structural recommendations

Recommendations featured under the theme of structural components of HRCB interventions related to overarching systemic issues of HRCB interventions. Studies recommended preparing for developing and implementing HRCB programmes by conducting a needs assessment in the FCAS of interest in order to ensure that the intervention is contextualized, relevant, and driven by the needs of the population [47, 55, 57]. Another suggestion was the preparation of a thorough approach or framework for the evaluation of HRCB activities that includes assessing a broad group of stakeholders, behavioural change, and additional long-term outcomes [40, 43, 51, 53, 55–57]. Further recommendations included taking a multidisciplinary system approach when developing HRCB initiatives by involving different health sectors and by targeting a broad range of stakeholders such as individual researchers and local institutions and research bodies [42, 50]. Studies also highlighted the need for greater equity in partnership involvement through bidirectional exchanges of staff from and to FCASs and partnering HICs, as well as more equitable opportunities for authorship as a result of HRCB activities [40, 44–46, 50, 51, 53]. For example, one study drafted by an HIC researcher and reporting on a mutually beneficial HRCB experience, reflected that their role as first author “epitomizes the unequal power, educational opportunity, language in which the publication is written and formal writing capacity that still lies with the most resourced, despite efforts to date” [57].

##### Content recommendations

With regard to the content of HRCB interventions theme, reviewed studies commonly recommended the design of programmes that deliver contextually relevant material through practical approaches, and to incorporate a mentorship angle to them. Studies reported that it was important for future initiatives to focus on material that incorporated issues of local relevance, such as through aligning the content of the training with health issues prioritized on the national- and regional-level agendas [41, 42, 54, 55]. In addition, through incorporating a mentorship component and through using practical pedagogical approaches which are more conducive to active learning, participants would have more opportunities for hands-on experiences and may feel more engaged with the learning material [41, 42, 50, 52, 54].

## Discussion

The topic of HRCB has been described broadly in a narrative review conducted by researchers in the R4HC-MENA consortium where they reflected on lessons learned from LMIC settings, and subsequently recommended strategies for HRCB programmes in FCASs [12]. The review was then followed by a paper presenting the first conceptual framework for HRCB initiatives designed for conflict settings [10]. However, to the best of our knowledge, the present study is the first scoping review which systematically maps and identifies the evidence in academic outlets regarding implemented HRCB interventions targeting health workers in FCASs.

Understandably, and potentially due to the fact that this field has only until recently surfaced [59], only 20 studies were identified since 2010, most of which were published after 2015. This highlights a significant gap in the available evidence despite the growing interest in conducting and strengthening health research in FCASs. Indeed, the observed change in the nature of contemporary conflicts, being more intrastate (proxy wars) rather than interstate, along with being protracted (average of 12 years) [60], has created a shift in paradigm from humanitarian short-termism, which is not fit for purpose anymore, into sustainable development [61]. This enhances the focus on strengthening local capacities at the individual, organizational, institutional, and system levels in order to bridge the gap between research, practice, and policy with the goal of having contextualized and impactful interventions in low-resource settings [62–64].

That being said, one could argue that the number of papers found in the academic literature does not reflect the actual number of ongoing and previously implemented HRCB interventions in FCASs given that most nongovernmental organizations (NGOs) prefer to communicate their findings in the form of reports, and often to donors only. This highlights one of the major challenges in health research, especially in conflict settings, namely, the lack of communication between academic and humanitarian sectors as they tend to work in silos. Ultimately, this leads to a loss in opportunities to avoid duplication of effort as well as to combine resources to produce local knowledge and design interventions tailored to local needs [65].

Surprisingly, only one study was reported from the MENA region by researchers in Lebanon, despite the fact that this region has continuously been plagued with protracted conflicts since as early as 1948. It also continues to host the worst humanitarian crises since the Second World War with almost 37% of 70.8 million people displaced worldwide originating from the region [66]. This is alarming because when considering the scale of protracted conflicts, displaced individuals, and the escalating health needs of the region’s population [67, 68], much more effort should be made to improve capacity in health research to influence policy and improve health outcomes. Given that there are a few ongoing projects, including R4HC-MENA, RECAP, and Center For Research and Education in the Ecology of War (CREEW) [29, 69, 70], all of which are focusing on HRCB in FCASs in the MENA region, it is likely to see more literature related to the topic of this review focusing on this region in the near future. In this review, most of the studies were concentrated in Africa, which is expected since most FCASs as defined by the World Bank are African countries. Also, Nigeria in specific produced the most research on HRCB, which is reassuring considering the conflicts the country endured and their impact on socioeconomic lives of people and their health system. A policy brief out of the Peace Research Institute Oslo importantly highlighted that there has been a continued increase in the number of conflicts in Africa, including state-based conflicts, non-state conflicts, and one-sided violence, due to an increase in the number of actors involved in the conflicts [71]. This has undoubtedly been reported to take a toll on millions of civilians. Consequences include being uprooted from homes, loss of livelihood, and increased violence and abuse against civilians [72]. It is thus essential that HRCB initiatives continue to take place in such settings in order to contribute to the strengthening of the fragile healthcare systems in place. It is noteworthy to also mention that as highlighted in Table 3, HRCB initiatives conducted in Africa had on average a greater number of participants compared to those in the Caribbean, Pacific Islands, or Middle East.

Another significant finding supported by previous studies [12, 22] is that the majority of published papers, as demonstrated by corresponding and first authorship, were led by authors affiliated primarily with institutions from the Global North rather than local authors. Relatedly, more than half of the studies reviewed were funded by government agencies or universities from the Global North. This demonstrates how power dynamics related to funding, colonial history, and human resources, may impact the location of decision-making and consequently direct and shape capacity building interventions and their dissemination. This is understandable given that the Global North hosts most of the reputable academic and global health centres involved in research within FCASs. Indeed, research institutes located in the Global South and working on issues of global health did not exist until recently. One example is the Global Health Institute (GHI) at the American University of Beirut (AUB) in Lebanon, which was established in 2017 and which is considered the first of its kind in the MENA region and among the very few in the Global South [73, 74]. Being aware of this discordance, and in an attempt to mitigate it, major funding agencies are currently requesting for extra measures to be implemented to ensure equitable and effective interventions and north-south partnerships [10, 75–79]. This is because locally led initiatives have a deeper understanding of local context and are proving to resonate better with local needs, knowledge, and narratives, all of which may have been otherwise neglected in favour of global unitary knowledge set by the Global North [80].

Despite the number of studies included in this review being too small to generalize, several points were highlighted regarding the strengths and weaknesses of the reported HRCB interventions. As an example, most strengths reported in the studies highlighted that the capacity building intervention was based on a needs assessment and/or context-specific design in order to ensure local relevance of the programme. This falls in line with major requirements of any capacity building initiative, especially in conflict and ongoing war settings where humanitarian agencies tend to conduct rapid needs assessments to guide their efforts [81–85]. However, despite this being a strength of most initiatives, it would be useful in future efforts to explore the stage at which the needs assessment was conducted and specifically if it was performed before or after the funding was granted, in order to determine whether the project as a whole and/or the topic of the training was predetermined by the funding body. This is important considering that research waste has recently been reported in FCASs, such as in the MENA region, although most of the funded projects were presumably “needs-oriented” [86–88]. Moreover, although linking the capacity building intervention to the local burden of disease may be ideal, it will also be challenging, as the lack of reliable data in FCASs is endemic and because in such settings, health data is often securitized and politically charged [80, 89–91]. Additional strengths were identified from the reviewed studies. For example, programmes that were interactive and hands-on, which offered increased practicality to participants, and which gave them the opportunity to have an experiential learning process, were reported as being effective. Also, HRCB programmes that included multidisciplinary participation and that were holistic in nature were reported to be beneficial.

With regard to the weaknesses, most of the reviewed papers reflected short-term and generic descriptions of a given intervention with little to no report on short- or long-term evaluations or impact assessments. Although the political and social instability throughout FCASs prompts the implementation of short-term interventions, a follow-up strategy for assessing the feasibility, benefits, and impacts of such interventions is crucial as it informs future directions and contributes to the sustainability of capacity building projects [92, 93]. The problem of sustainability for research in FCASs is primarily a matter of resources, as demonstrated by the major disparity in spending on research between HIC and LMICs. For instance, according to the United Nations Educational, Scientific and Cultural Organization (UNESCO) Institute for Statistics, HICs spent US $1.5 trillion on research and development in 2018, which is 16 times as much as LMICs have spent, and more than 400 times as much as LICs. Thus, to improve the sustainability of projects, international agencies such as WHO should support identification, establishment, and development of health research centres in LMICs to create a network of centres that can share resources and allocate funding to high-priority health system needs, including for research on capacity building.

In addition, almost all of the reviewed initiatives focused on HRCB at the individual level. Despite the importance of focusing on building the capacity of individuals, and although all levels (individual, organizational, institutional, and systemic) are highly interconnected, and that strengthening one level will automatically strengthen the other three levels, it is important to note that FCASs suffer from high levels of staff turnover due to brain drain and injury/death. For example, 96% of Syrian health workers living in Aleppo fled the city as of 2016 [94]. As such, focusing solely on building individual research capacity may be considered less sustainable when compared to investing in strengthening organizational and institutional research capacity. This is crucial since the latter may eventually decrease brain drain and ameliorate major challenges such as by providing safely accessible infrastructure, reliable data and databases, and a permissive environment.

Furthermore, the reviewed studies did not employ a gender-sensitive approach and did not consider gender equity in their interventions; in fact, only three studies reported the gender of their participants [41, 43, 58]. Despite that gender inequity is a problem reported at the global scale, women are disproportionately affected by conflict and fragility [95]. As an example, it has been repeatedly documented in the literature that most FCASs struggle with entrenched cultural, social, and political gender discrimination [96–98]. Therefore, introducing a gender lens to future HRCB programmes would be ideal since it helps in normalizing gender equity, particularly across conflict settings. It has been shown that empowering women can transform systems to better meet a populations’ health needs, specifically within marginalized communities, and can provide broader understanding of the global health system which is urgently needed for the ongoing transfer in paradigm from short-termism to sustainable development of health systems in conflict [99–101].

Finally, findings from our qualitative analysis align well with previous studies in FCASs like Lebanon and Palestine [65, 102, 103]. The lack of nationwide research culture, insufficient funding, poor impact of research on policy, and limited access to data were all reported to be major challenges in FCASs and for implementing HRCB programmes [61]. However, additional concerns were also expressed in prior papers regarding the ethics of research conducted, specifically by local NGOs [65]. This topic was only addressed in a few of the selected academic papers despite its importance. This is particularly true given that Western concepts of confidentiality and individualism may not fit with collectivistic cultures and other settings.

The aforementioned findings should be interpreted in light of some limitations. For instance, the fact that HRCB is a field still relatively in its infancy limited the number of studies we were able to find throughout our search, which in turn reduced our ability to generalize our findings. On that note, we may have encountered publication bias since we did not include grey literature record and restricted our search to only electronic databases. Additionally, the small sample size made it challenging for us to compare the research topics addressed in the included studies with regional- or country-level health needs and subsequently make specific recommendations. Methodologically, some bias may have ensued during the extraction of the data since only one reviewer completed this process; nevertheless, the instrument used in this process was piloted, and internal discussions were had among team members regarding its validity in relation to the study aims. Also, the search was based on a systematic process using keywords that align with our definition of capacity building, in a field with inconsistent terminologies and unstandardized key terms [104]. This may have caused some papers not to be detected; however, we made sure to include all possible terms in the search strategy. Finally, we only included articles written in English, and we may have thus missed articles written in Arabic, French, or other local languages.

## Conclusion and recommendations

This paper represents the first scoping review of HRCB in FCASs. Despite it being a relatively novel field, we have summarized and analysed the characteristics of HRCB efforts conducted over the past decade, along with their major strengths and weaknesses. Our findings funnel into key recommendations for related policy-makers, institutions, and health personnel. Overall, there is a strong need for:

1. FCASs to allocate local funding for HRCB programmes, to equip these programmes with adequate human and material resources, and to lead their own projects in order to reduce dependence on institutions from the global north;
2. HRCB programmes to equip trainers with an understanding of the specific needs and cultural nuances of the local context;
3. FCASs to design, implement, and evaluate long-term HRCB programmes that address the organizational, institutional, and system levels in addition to the individual level in order to enhance the impact and sustainability of HRCB efforts;
4. HRCB programmes to be developed and delivered through local, regional, and international partnerships;
5. HRCB programmes to be contextually relevant, and to be delivered using engaging and practical hands-on approaches.

## Supplementary Information

**Additional file 1: Appendix 1.** Search strategy**.**

**Additional file 2: Appendix 2.** List of categories, themes, codes, as well as the number of studies that included each code based on thematic analysis of studies included in the scoping review.

## Data Availability

All data generated or analysed during this study are included in this published article [and its supplementary information files].

## Abbreviations

LMIC: Low- and middle-income countries
FCAS: Fragile and conflict-affected settings
HIC: High-income country
LIC: Low-income country
HRCB: Health research capacity building
FETP: Field epidemiology training programme
CDC: Centers for Disease Control and Prevention
EIS: Epidemic Intelligence Services
R4HC-MENA: Research for Health in Conflict MENA
MENA: Middle East and North Africa
AUB: American University of Beirut
GHI: Global Health Institute
CREEW: Center for Research and Education in the Ecology of War
RECAP: Research capacity-strengthening and knowledge generation to support preparedness and response to humanitarian crises and epidemics
