# Supplementary material for: Health research capacity building of health workers in fragile and conflict-affected settings: a scoping review of challenges, strengths, and recommendations
Source: Health Res Policy Syst. 2021 May 22;19:84. doi: 10.1186/s12961-021-00725-x (PMC8140497; doi:10.1186/s12961-021-00725-x)
Supplement: Supplementary file 1 — Additional file 1: Appendix 1. Search strategy. [file 12961_2021_725_MOESM1_ESM.docx]

**Supplemental Appendix 1: Search Strategy**

Our final search was conducted on May 4, 2020. Four databases were searched with the following strategies:

**Database: COCHRANE**

Search Name: Capacity Building+Research+healthcare personel+LMIC

Date Run: 04/05/2020 13:07:59

Comment:

--------------------------------------------------------------------------------

ID Search Hits

#1 MeSH descriptor: [Capacity Building] this term only 31

#2 (capacity NEAR/2 building):ti,ab,kw 245

#3 MeSH descriptor: [Education, Distance] this term only 143

#4 MeSH descriptor: [Health Promotion] this term only 5592

#5 MeSH descriptor: [Education] this term only 571

#6 MeSH descriptor: [Webcast] this term only 0

#7 MeSH descriptor: [Videoconferencing] explode all trees 195

#8 MeSH descriptor: [Educational Technology] this term only 63

#9 MeSH descriptor: [Learning] this term only 2131

#10 MeSH descriptor: [Teaching] this term only 1696

#11 (Course* or Webinar* or e-learning or elearning or learn* or online-learn* or teach* or teach-back or educat* or ((healthcare or health*) NEAR/1 (information or promot*)) or ((program or health) NEAR/3 litera*) or ((health or wellness or healthcare or health-care or smok* or (anti NEXT/0 smok*)) NEAR/3 (promotion* or campaign* or awareness or programs)) or workshop* or (promotion* NEAR/3 item*) or (health NEAR/3 fair*) or MOOCs or train* or ToT or (Pedagogic* NEAR/1 approach*) or webcast* or tutorial* or (discussion NEXT/0 (forum or board)) or videoconferenc* or video-conferenc* or moodle or blackboard):ti,ab,kw 241338

#12 MeSH descriptor: [Teaching Materials] this term only 425

#13 MeSH descriptor: [Program Development] this term only 697

#14 (program NEXT/1 (development* OR description*)):ti,ab,kw 971

#15 MeSH descriptor: [Fellowships and Scholarships] this term only 25

#16 (Fellowship* OR Scholarship*):ti,ab,kw 449

#17 (global NEAR/2 (intervention* OR initiative*)):ti,ab,kw 712

#18 #1 OR #2 OR #3 OR #4 OR #5 OR #6 OR #7 OR #8 OR #9 OR #10 #12 #13 OR #14 OR #15 OR #16 OR #17 10765

#19 (((((medical OR nurs* OR dental) NEXT/1 (student* OR graduate* OR intern OR Interns OR fellow OR fellows)) OR (labor NEAR/3 coach*) OR md OR anatomist* OR audiologist* OR coroner* OR ((health* OR hospital OR medical OR clinic* OR inpatient* OR outpatient*) NEAR/3 (examiner* OR assistant* OR administrator* OR supervisor* OR receptionist* OR personnel OR team OR staff OR worker* OR researcher OR researchers OR librarian* OR informationist* OR technician* OR manager* OR housekeeper* OR janitor* OR officer* OR responder* OR paramedic* OR laboratorian* OR coordinator* OR navigator* OR attendant* OR scientist* OR cashier* OR accountant* OR registrar OR registrars OR provider* OR chaperone OR chaperones OR man-power OR manpower OR workforce* OR caregiver*)) OR ((er OR emergency OR “911”) NEXT/1 dispatcher*) OR epidemiologist* OR midwife* OR midwive* OR (medical NEXT/1 care NEXT/1 giver*) OR (hospital NEXT/1 care NEXT/1 giver*) OR (clinical NEXT/1 care NEXT/1 giver*) OR (house NEXT/1 officer*) OR resident OR residents OR nurse OR nurses OR nursing OR orderly OR orderlies OR dietitian* OR dietician* OR psychologist* OR counselor* OR therapist* OR pharmacist* OR hygeinist* OR phlebotomist* OR chiropracter* OR acupuncturist* OR anesthesiologist* OR anaesthesiologist* OR anesthetist* OR anaesthetist* OR perfusionist* OR surgeon* OR pediatrician* OR paediatrician* OR obstetrician* OR gynecologist* Or gynaecologist* OR radiologist* OR sonographer* OR cardiologist* OR gastroenterologist* OR hepatologist* OR endocrinologist* OR diabetologist* OR internist* OR hospitalist* OR intensivist* OR rheumatologist* OR immunologist* OR dermatologist* OR allergist* OR otolaryngologist* OR otorhinolaryngologist* OR oncologist* OR hematologist* OR haematologist* OR orthopedist* OR orthopaedist* OR physiatrist* OR podiatrist* OR pathologist* OR diener* OR cytologist* OR cytogeneticist* OR geneticist* OR hematopathologist* OR haematopathologist* OR neurologist* OR neurosurgeon* OR ophthalmologist* OR optician* OR urologist* OR nephrologist* OR pulmonologist* OR neonatologist* OR psychiatrist* OR physiotherapist* OR dentist* OR prosthodontist* OR periodontist* OR endodontist OR nutritionist* OR optometrist* OR geriatrician* OR gerontologist* OR otologist* OR feldsher* OR exodontist* OR doula*))):ti,ab,kw 137994

#20 MeSH descriptor: [Health Personnel] explode all trees 8495

#21 MeSH descriptor: [Students, Health Occupations] explode all trees 1607

#22 MeSH descriptor: [Health Workforce] this term only 17

#23 #19 OR #20 OR #21 OR #22 139991

#24 MeSH descriptor: [Dual Use Research] this term only 0

#25 MeSH descriptor: [Research Subjects] explode all trees 3363

#26 MeSH descriptor: [Genetic Research] this term only 6

#27 MeSH descriptor: [Research] explode all trees 19671

#28 MeSH descriptor: [Research Support as Topic] this term only 39

#29 MeSH descriptor: [Ethics, Research] this term only 43

#30 MeSH descriptor: [Stem Cell Research] this term only 0

#31 ((Research* or (medicine NEAR/2 investigat*) or ((Health* or retrospective or cohort or prospective) and (Volunteer? or participant?)))):ti,ab,kw 260687

#32 #24 OR #25 OR #26 OR #27 OR #28 OR #29 OR #30 OR #31 264657

#33 ((Afghanistan* or (Central NEXT African NEXT Republic) or Libya* or Somalia* or (South NEXT Sudan*) or Syria* or syrie* or Yemen* or (Burkina NEXT Faso*) or Burundi* or Cameroon* or Congo* or Iraq* or irak* or Mali* or Niger* or Nigeria* or Sudan* or Chad* or Eritrea* or Gambia* or Guinea-Bissau* or Haiti* or Kosovo* or Leban* or liban* or l?bnan* or Liberia* or Myanmar* or (Papua NEXT New NEXT Guinea*) or Venezuel* or Zimbabw* or Comoros or Kiribati* or (Marshall NEXT Island*) or Micronesia* or Timor-Leste or Tuvalu or palestin* or ghazza or ghaza or gaza or (west* NEAR/2 bank) or (Solomon NEXT Islands))):ti,ab,kw 32539

#34 MeSH descriptor: [Afghanistan] this term only 39

#35 MeSH descriptor: [Central African Republic] this term only 12

#36 MeSH descriptor: [Libya] this term only 6

#37 MeSH descriptor: [Somalia] this term only 18

#38 MeSH descriptor: [South Sudan] this term only 0

#39 MeSH descriptor: [Syria] this term only 25

#40 MeSH descriptor: [Yemen] this term only 5

#41 MeSH descriptor: [Burkina Faso] this term only 152

#42 MeSH descriptor: [Burundi] this term only 11

#43 MeSH descriptor: [Cameroon] this term only 99

#44 MeSH descriptor: [Congo] this term only 13

#45 MeSH descriptor: [Democratic Republic of the Congo] this term only 91

#46 MeSH descriptor: [Iraq] this term only 48

#47 MeSH descriptor: [Mali] this term only 99

#48 MeSH descriptor: [Niger] this term only 52

#49 MeSH descriptor: [Nigeria] this term only 561

#50 MeSH descriptor: [Sudan] this term only 83

#51 MeSH descriptor: [Chad] this term only 5

#52 MeSH descriptor: [Eritrea] this term only 1

#53 MeSH descriptor: [Gambia] this term only 226

#54 MeSH descriptor: [Guinea-Bissau] this term only 91

#55 MeSH descriptor: [Haiti] this term only 57

#56 MeSH descriptor: [Kosovo] this term only 3

#57 MeSH descriptor: [Lebanon] this term only 57

#58 MeSH descriptor: [Liberia] this term only 22

#59 MeSH descriptor: [Myanmar] this term only 63

#60 MeSH descriptor: [Papua New Guinea] this term only 58

#61 MeSH descriptor: [Venezuela] this term only 51

#62 MeSH descriptor: [Zimbabwe] this term only 193

#63 MeSH descriptor: [Comoros] this term only 1

#64 MeSH descriptor: [Micronesia] this term only 6

#65 MeSH descriptor: [Micronesia] this term only 6

#66 MeSH descriptor: [Timor-Leste] this term only 4

#67 MeSH descriptor: [Micronesia] this term only 6

#68 MeSH descriptor: [Melanesia] this term only 5

#69 #33 OR #34 OR #35 OR #36 OR #37 OR #38 OR #39 OR #40 OR #41 OR #42 OR #43 OR #44 OR #45 OR #46 OR #47 OR #48 OR #49 OR #50 OR #51 OR #52 OR #53 OR #54 OR #55 OR #56 OR #57 OR #58 OR # 59 OR #60 OR #61 OR #62 OR #63 OR #64 OR #65 OR #66 OR #67 OR #68 98324

#70 #18 AND #23 AND #32 AND #69 114

**Database: MEDLINE**

Database: Ovid MEDLINE(R) and Epub Ahead of Print, In-Process & Other Non-Indexed Citations and Daily <1946 to April 24, 2020>

Search Strategy:

--------------------------------------------------------------------------------

1 Capacity Building/ (2458)

2 (capacity adj2 building).mp. (7107)

3 education, distance/ or health promotion/ or healthy people programs/ or mentoring/ or exp Education, Nonprofessional/ or Education/ or Webcasts/ or exp videoconferencing/ or Educational Technology/ or Learning/ or Teaching/ or Pamphlets/ (397182)

4 (Course? or Webinar? or e-learning or elearning or learn* or online-learn* or teach* or teach-back or educat* or ((healthcare or health*) adj3 (information or promot*)) or ((program or health) adj3 litera*) or ((health or wellness or healthcare or health-care or smok* or (anti adj smok*)) adj3 (promotion* or campaign? or awareness or programs)) or workshop? or (promotion* adj3 item?) or (health adj3 fair?) or MOOCs or train* or ToT or (Pedagogic* adj3 approach*) or webcast* or tutorial* or (discussion adj (forum or board)) or Pamphlet* or brochur* or hand-out? or handout? or booklet? or videoconferenc* or video-conferenc* or moodle or blackboard).mp. (2506297)

5 Teaching Materials/ (6416)

6 Program Development/ (28727)

7 (program adj (development? or description?)).mp. (31180)

8 "Fellowships and Scholarships"/ or (Fellowship? or Scholarship? or (global adj2 (intervention* or initiative?))).mp. (23568)

9 (((access or freedom) adj3 (open or information)) or (FOIA adj Request?)).mp. or Access to Information/ (21618)

10 Information Dissemination/ or ((information or data) adj2 (sharing* or disseminat* or distribut*)).mp. (36478)

11 Problem Solving/ or (problem adj3 solv*).mp. (55432)

12 or/1-11 (2613620)

13 (((health* or hospital or medical or clinic* or inpatient* or outpatient*) adj3 (examiner* or assistant? or administrator* or supervisor* or receptionist* or personnel or team or staff or worker* or researcher or researchers or librarian* or informationist* or technician* or manager* or housekeeper* or janitor* or officer* or responder* or paramedic* or laboratorian* or coordinator* or navigator* or attendant* or scientist* or cashier? or accountant? or registrar? or provider* or chaperone? or man-power or manpower or workforce* or (care adj giver*) or caregiver?)) or epidemiologist* or ((ER or emergency or "911") adj dispatcher*) or midwi?e?).mp. (513278)

14 (physician* or doctor* or allopath? or osteopath? or homeopath? or ((clinical* or health* or medical) adj (practitioner? or specialist? or generalist?)) or ((medical or nurs* or dental) adj (student? or graduate? or intern? or fellow?)) or (house adj officer?) or Resident? or nurse? or nursing or orderly or orderlies or dieti?ian* or psychologist* or counselor* or therapist* or pharmacist* or dentist* or hygeinist* or phlebotomist* or chiropracter* or acupuncturist* or an?esthesiologist* or an?esthetist* or perfusionist* or surgeon* or p?ediatrician* or obstetrician* or gyn?ecologist* or radiologist* or sonographer* or cardiologist* or gastroenterologist* or hepatologist* or endocrinologist* or diabetologist* or internist* or hospitalist* or intensivist* or rheumatologist* or immunologist* or dermatologist* or allergist* or otolaryngologist* or otorhinolaryngologist* or oncologist* or h?ematologist* or orthop?edist* or physiatrist* or podiatrist* or pathologist* or diener* or cytologist* or cytogeneticist* or geneticist* or h?ematopathologist* or neurologist* or neurosurgeon* or ophthalmologist* or optician* or urologist* or nephrologist* or pulmonologist* or neonatologist* or psychiatrist* or physiotherapist* or dentist* prosthodontist* or periodontist* or endodontist or nutritionist* or optometrist* or geriatrician* or gerontologist* or otologist* or feldsher* or exodontist* or doula* or (labor adj3 coach*) or MD or anatomist* or audiologist* or coroner?).mp. (2138835)

15 exp Health Personnel/ or exp Students, Health Occupations/ or health workforce/ (571058)

16 13 or 14 or 15 (2444651)

17 "Dual Use Research"/ or exp Research Subjects/ or Genetic Research/ or exp Research/ or Research Support as Topic/ or Ethics, Research/ or Stem Cell Research/ (646445)

18 (Research* or (medicine adj2 investigat*) or ((Health* or retrospective or cohort or prospective) and (Volunteer? or participant?))).mp. (10578529)

19 17 or 18 (10622113)

20 Afghanistan/ or Central African Republic/ or Libya/ or Somalia/ or South Sudan/ or Syria/ or Yemen/ or Burkina Faso/ or Burundi/ or Cameroon/ or Congo/ or "Democratic Republic of the Congo"/ or Iraq/ or Mali/ or Niger/ or Nigeria/ or Sudan/ or Chad/ or Eritrea/ or Gambia/ or Guinea-Bissau/ or Haiti/ or Kosovo/ or Lebanon/ or Liberia/ or Myanmar/ or Papua New Guinea/ or Venezuela/ or Zimbabwe/ or Comoros/ or Kiribati/ or Micronesia/ or Timor-Leste/ or Tuvalu/ or Melanesia/ (94868)

21 (Afghanistan* or (Central adj African adj Republic) or Libya* or Somalia* or (South adj Sudan*) or Syria* or syrie* or Yemen* or (Burkina adj Faso*) or Burundi* or Cameroon* or Congo* or Iraq* or irak* or Mali* or Niger* or Nigeria* or Sudan* or Chad* or Eritrea* or Gambia* or Guinea-Bissau* or Haiti* or Kosovo* or Leban* or liban* or l?bnan* or Liberia* or Myanmar* or (Papua adj New adj Guinea*) or Venezuel* or Zimbabw* or Comoros or Kiribati* or (Marshall adj Island*) or Micronesia* or Timor-Leste or Tuvalu or palestin* or ghazza or ghaza or gaza or (west* adj2 bank) or (Solomon adj Islands)).mp. (780429)

22 20 or 21 (780869)

23 12 and 16 and 19 and 22 (6425)

***************************

**Database: CINAHL**

Friday, May 01, 2020 4:33:34 AM

| **#** | **Query** | **Limiters/Expanders** | **Last Run Via** | **Results** |
| --- | --- | --- | --- | --- |
| S11 | S3 AND S4 AND S7 AND S10 | Expanders - Apply equivalent subjects  Search modes - Boolean/Phrase | Interface - EBSCOhost Research Databases  Search Screen - Advanced Search  Database - CINAHL Complete | 4,790 |
| S10 | S8 OR S9 | Expanders - Apply equivalent subjects  Search modes - Boolean/Phrase | Interface - EBSCOhost Research Databases  Search Screen - Advanced Search  Database - CINAHL Complete | 120,157 |
| S9 | (MH "Libya") OR (MH "Congo") OR (MH "Democratic Republic of the Congo") OR (MH "Central African Republic") OR (MH "Afghanistan") OR (MH "Somalia") OR (MH "Syria") OR (MH "Yemen") OR (MH "Burkina Faso") OR (MH "Burundi") OR (MH "Cameroon") OR (MH "Iraq") OR (MH "Mali") OR (MH "Niger") OR (MH "Nigeria") OR (MH "Chad") OR (MH "Eritrea") OR (MH "Gambia") OR (MH "Guinea-Bissau") OR (MH "Haiti") OR (MH "Lebanon") OR (MH "Liberia") OR (MH "Myanmar") OR (MH "Venezuela") OR (MH "Zimbabwe") | Expanders - Apply equivalent subjects  Search modes - Boolean/Phrase | Interface - EBSCOhost Research Databases  Search Screen - Advanced Search  Database - CINAHL Complete | 28,371 |
| S8 | TI ( (Afghanistan* OR (Central W0 African W0 Republic) OR Libya* OR Somalia* OR (South W0 Sudan*) OR Syria* OR syrie* OR Yemen* OR (Burkina N Faso*) OR Burundi* OR Cameroon* OR Congo* OR Iraq* OR irak* OR Mali* OR Niger* OR Nigeria* OR Sudan* OR Chad* OR Eritrea* OR Gambia* OR Guinea-Bissau* OR Haiti* OR Kosovo* OR Leban* OR liban* OR l#bnan* OR Liberia* OR Myanmar* OR (Papua W0 New W0 Guinea*) OR Venezuel* OR Zimbabw* OR Comoros OR Kiribati* OR (Marshall W0 Island*) OR Micronesia* OR Timor-Leste OR Tuvalu OR palestin* OR ghazza OR ghaza OR gaza OR (west* N1 bank) OR (Solomon W0 Islands)) ) OR AB ( (Afghanistan* OR (Central W0 African W0 Republic) OR Libya* OR Somalia* OR (South W0 Sudan*) OR Syria* OR syrie* OR Yemen* OR (Burkina N Faso*) OR Burundi* OR Cameroon* OR Congo* OR Iraq* OR irak* OR Mali* OR Niger* OR Nigeria* OR Sudan* OR Chad* OR Eritrea* OR Gambia* OR Guinea-Bissau* OR Haiti* OR Kosovo* OR Leban* OR liban* OR l#bnan* OR Liberia* OR Myanmar* OR (Papua W0 New W0 Guinea*) OR Venezuel* OR Zimbabw* OR Comoros OR Kiribati* OR (Marshall W0 Island*) OR Micronesia* OR Timor-Leste OR Tuvalu OR palestin* OR ghazza OR ghaza OR gaza OR (west* N1 bank) OR (Solomon W0 Islands)) ) OR MW ( (Afghanistan* OR (Central W0 African W0 Republic) OR Libya* OR Somalia* OR (South W0 Sudan*) OR Syria* OR syrie* OR Yemen* OR (Burkina N Faso*) OR Burundi* OR Cameroon* OR Congo* OR Iraq* OR irak* OR Mali* OR Niger* OR Nigeria* OR Sudan* OR Chad* OR Eritrea* OR Gambia* OR Guinea-Bissau* OR Haiti* OR Kosovo* OR Leban* OR liban* OR l#bnan* OR Liberia* OR Myanmar* OR (Papua W0 New W0 Guinea*) OR Venezuel* OR Zimbabw* OR Comoros OR Kiribati* OR (Marshall W0 Island*) OR Micronesia* OR Timor-Leste OR Tuvalu OR palestin* OR ghazza OR ghaza OR gaza OR (west* N1 bank) OR (Solomon W0 Islands)) ) | Expanders - Apply equivalent subjects  Search modes - Boolean/Phrase | Interface - EBSCOhost Research Databases  Search Screen - Advanced Search  Database - CINAHL Complete | 119,470 |
| S7 | S5 OR S6 | Expanders - Apply equivalent subjects  Search modes - Boolean/Phrase | Interface - EBSCOhost Research Databases  Search Screen - Advanced Search  Database - CINAHL Complete | 3,115,720 |
| S6 | (MH "Research Subjects+") OR (MH "Genetic Research+") OR (MH "Research+") OR (MH "Research Ethics+") OR (MH "Stem Cell Research") OR (MH "Research, Medical") | Expanders - Apply equivalent subjects  Search modes - Boolean/Phrase | Interface - EBSCOhost Research Databases  Search Screen - Advanced Search  Database - CINAHL Complete | 2,853,226 |
| S5 | TI ( (Research* OR (medicine N1 investigat*) OR ((Health* OR retrospective OR cohort OR prospective) AND (Volunteer# OR participant#))) ) OR AB ( (Research* OR (medicine N1 investigat*) OR ((Health* OR retrospective OR cohort OR prospective) AND (Volunteer# OR participant#))) ) OR MW ( (Research* OR (medicine N1 investigat*) OR ((Health* OR retrospective OR cohort OR prospective) AND (Volunteer# OR participant#))) ) | Expanders - Apply equivalent subjects  Search modes - Boolean/Phrase | Interface - EBSCOhost Research Databases  Search Screen - Advanced Search  Database - CINAHL Complete | 1,321,214 |
| S4 | (MH "Health Personnel+") OR (MH "Students, Health Occupations+") OR TI (physician* OR doctOR* OR allopath# OR osteopath# OR homeopath# OR ((clinical* OR health* OR medical) W1 (practitioner# OR specialist# OR generalist#)) OR ((medical OR nurs* OR dental) W1 (student# OR graduate# OR intern# OR fellow#)) OR (house W1 officer#) OR Resident# OR nurse# OR nursing OR ORderly OR ORderlies OR dieti#ian* OR psychologist* OR counselOR* OR therapist* OR pharmacist* OR dentist* OR hygeinist* OR phlebotomist* OR chiropracter* OR acupuncturist* OR an#esthesiologist* OR an#esthetist* OR perfusionist* OR surgeon* OR p#ediatrician* OR obstetrician* OR gynaecologist* OR gynecologist* OR radiologist* OR sonographer* OR cardiologist* OR gastroenterologist* OR hepatologist* OR endocrinologist* OR diabetologist* OR internist* OR hospitalist* OR intensivist* OR rheumatologist* OR immunologist* OR dermatologist* OR allergist* OR otolaryngologist* OR otORhinolaryngologist* OR oncologist* OR h#ematologist* OR ORthop#edist* OR physiatrist* OR podiatrist* OR pathologist* OR diener* OR cytologist* OR cytogeneticist* OR geneticist* OR h#ematopathologist* OR neurologist* OR neurosurgeon* OR ophthalmologist* OR optician* OR urologist* OR nephrologist* OR pulmonologist* OR neonatologist* OR psychiatrist* OR physiotherapist* OR dentist* OR prosthodontist* OR periodontist* OR endodontist OR nutritionist* OR optometrist* OR geriatrician* OR gerontologist* OR otologist* OR feldsher* OR exodontist* OR doula* OR (labOR N3 coach*) OR MD OR anatomist* OR audiologist* OR cORoner#) OR AB (physician* OR doctOR* OR allopath# OR osteopath# OR homeopath# OR ((clinical* OR health* OR medical) W1 (practitioner# OR specialist# OR generalist#)) OR ((medical OR nurs* OR dental) W1 (student# OR graduate# OR intern# OR fellow#)) OR (house W1 officer#) OR Resident# OR nurse# OR nursing OR ORderly OR ORderlies OR dieti#ian* OR psychologist* OR counselOR* OR therapist* OR pharmacist* OR dentist* OR hygeinist* OR phlebotomist* OR chiropracter* OR acupuncturist* OR an#esthesiologist* OR an#esthetist* OR perfusionist* OR surgeon* OR p#ediatrician* OR obstetrician* OR gyn#ecologist* OR radiologist* OR sonographer* OR cardiologist* OR gastroenterologist* OR hepatologist* OR endocrinologist* OR diabetologist* OR internist* OR hospitalist* OR intensivist* OR rheumatologist* OR immunologist* OR dermatologist* OR allergist* OR otolaryngologist* OR otORhinolaryngologist* OR oncologist* OR h#ematologist* OR ORthop#edist* OR physiatrist* OR podiatrist* OR pathologist* OR diener* OR cytologist* OR cytogeneticist* OR geneticist* OR h#ematopathologist* OR neurologist* OR neurosurgeon* OR ophthalmologist* OR optician* OR urologist* OR nephrologist* OR pulmonologist* OR neonatologist* OR psychiatrist* OR physiotherapist* OR dentist* OR prosthodontist* OR periodontist* OR endodontist OR nutritionist* OR optometrist* OR geriatrician* OR gerontologist* OR otologist* OR feldsher* OR exodontist* OR doula* OR (labOR N3 coach*) OR MD OR anatomist* OR audiologist* OR cORoner#) OR TI (((health* OR hospital OR medical OR clinic* OR inpatient* OR outpatient*) N3 (examiner* OR assistant# OR administratOR* OR supervisOR* OR receptionist* OR personnel OR team OR staff OR wORker* OR researcher OR researchers OR librarian* OR infORmationist* OR technician* OR manager* OR housekeeper* OR janitOR* OR officer* OR responder* OR paramedic* OR labORatORian* OR coORdinatOR* OR navigatOR* OR attendant* OR scientist* OR cashier# OR accountant# OR registrar# OR provider* OR chaperone# OR man-power OR manpower OR wORkfORce* OR (care W1 giver*) OR caregiver#)) OR epidemiologist* OR ((ER OR emergency OR "911") W1 dispatcher*) OR midwi#e#) OR AB (((health* OR hospital OR medical OR clinic* OR inpatient* OR outpatient*) N3 (examiner* OR assistant# OR administratOR* OR supervisOR* OR receptionist* OR personnel OR team OR staff OR wORker* OR researcher OR researchers OR librarian* OR infORmationist* OR technician* OR manager* OR housekeeper* OR janitOR* OR officer* OR responder* OR paramedic* OR labORatORian* OR coORdinatOR* OR navigatOR* OR attendant* OR scientist* OR cashier# OR accountant# OR registrar# OR provider* OR chaperone# OR man-power OR manpower OR wORkfORce* OR (care W1 giver*) OR caregiver#)) OR epidemiologist* OR ((ER OR emergency OR "911") W1 dispatcher*) OR midwi#e#) | Expanders - Apply equivalent subjects  Search modes - Boolean/Phrase | Interface - EBSCOhost Research Databases  Search Screen - Advanced Search  Database - CINAHL Complete | 1,499,166 |
| S3 | S1 OR S2 | Expanders - Apply equivalent subjects  Search modes - Boolean/Phrase | Interface - EBSCOhost Research Databases  Search Screen - Advanced Search  Database - CINAHL Complete | 1,531,667 |
| S2 | (MH "Pamphlets") OR (MH "Teaching") OR (MH "Learning") OR (MH "Educational Technology") OR (MH "Videoconferencing+") OR (MH "Telenursing") OR (MH "Webcasts+") OR (MH "Education") OR (MH "Education, Nonprofessional") OR (MH "Mentorship") OR (MH "Healthy People 2020") OR (MH "Healthy People 2010") OR (MH "Healthy People 2000") OR (MH "Health Promotion") OR (MH "Education, Non-Traditional+") OR (MH "Problem Solving+") OR (MH "Scholarship") OR (MH "Training Support, Financial") OR (MH "Teaching Materials+") OR (MH "Selective Dissemination of Information") OR (MH "Access to Information+") | Expanders - Apply equivalent subjects  Search modes - Boolean/Phrase | Interface - EBSCOhost Research Databases  Search Screen - Advanced Search  Database - CINAHL Complete | 332,027 |
| S1 | TI ( (((access OR freedom) N2 (open OR information)) OR (FOIA W0 Request#)) OR (problem N2 solv*) OR ((information OR data) N1 (sharing* OR disseminat* OR distribut*)) OR (capacity W1 building) OR Course# OR Webinar# OR e-learning OR elearning OR learn* OR online-learn* OR teach* OR teach-back OR educat* OR ((healthcare OR health*) N2 (information OR promot*)) OR ((program OR health) N2 litera*) OR ((health OR wellness OR healthcare OR health-care OR smok* OR (anti W0 smok*)) N2 (promotion* OR campaign# OR awareness OR programs)) OR workshop# OR (promotion* N2 item#) OR (health N2 fair#) OR MOOCs OR train* OR ToT OR (Pedagogic* N2 approach*) OR webcast* OR tutorial* OR (discussion W0 (forum OR board)) OR Pamphlet* OR brochur* OR hand-out# OR handout# OR booklet# OR videoconferenc* OR video-conferenc* OR moodle OR blackboard) OR (program W0 (development# OR description#)) OR (Fellowship# OR Scholarship# OR (global N1 (intervention* OR initiative#))) OR (((access OR freedom) N2 (open OR information)) OR (FOIA W0 Request#)) ) OR AB ( (((access OR freedom) N2 (open OR information)) OR (FOIA W0 Request#)) OR (problem N2 solv*) OR ((information OR data) N1 (sharing* OR disseminat* OR distribut*)) OR (capacity W1 building) OR Course# OR Webinar# OR e-learning OR elearning OR learn* OR online-learn* OR teach* OR teach-back OR educat* OR ((healthcare OR health*) N2 (information OR promot*)) OR ((program OR health) N2 litera*) OR ((health OR wellness OR healthcare OR health-care OR smok* OR (anti W0 smok*)) N2 (promotion* OR campaign# OR awareness OR programs)) OR workshop# OR (promotion* N2 item#) OR (health N2 fair#) OR MOOCs OR train* OR ToT OR (Pedagogic* N2 approach*) OR webcast* OR tutorial* OR (discussion W0 (forum OR board)) OR Pamphlet* OR brochur* OR hand-out# OR handout# OR booklet# OR videoconferenc* OR video-conferenc* OR moodle OR blackboard) OR (program W0 (development# OR description#)) OR (Fellowship# OR Scholarship# OR (global N1 (intervention* OR initiative#))) OR (((access OR freedom) N2 (open OR information)) OR (FOIA W0 Request#)) ) OR MW ( (((access OR freedom) N2 (open OR information)) OR (FOIA W0 Request#)) OR (problem N2 solv*) OR ((information OR data) N1 (sharing* OR disseminat* OR distribut*)) OR (capacity W1 building) OR Course# OR Webinar# OR e-learning OR elearning OR learn* OR online-learn* OR teach* OR teach-back OR educat* OR ((healthcare OR health*) N2 (information OR promot*)) OR ((program OR health) N2 litera*) OR ((health OR wellness OR healthcare OR health-care OR smok* OR (anti W0 smok*)) N2 (promotion* OR campaign# OR awareness OR programs)) OR workshop# OR (promotion* N2 item#) OR (health N2 fair#) OR MOOCs OR train* OR ToT OR (Pedagogic* N2 approach*) OR webcast* OR tutorial* OR (discussion W0 (forum OR board)) OR Pamphlet* OR brochur* OR hand-out# OR handout# OR booklet# OR videoconferenc* OR video-conferenc* OR moodle OR blackboard) OR (program W0 (development# OR description#)) OR (Fellowship# OR Scholarship# OR (global N1 (intervention* OR initiative#))) OR (((access OR freedom) N2 (open OR information)) OR (FOIA W0 Request#)) ) | Expanders - Apply equivalent subjects  Search modes - Boolean/Phrase | Interface - EBSCOhost Research Databases  Search Screen - Advanced Search  Database - CINAHL Complete | 1,445,487 |

**Database: EMBASE**

| **No.** | **Query** | **Results** | **Date** |
| --- | --- | --- | --- |
| **#15** | #5 AND #8 AND #11 AND #14 | 6494 | 4 May 2020 |
| **#14** | #12 OR #13 | 1051876 | 4 May 2020 |
| **#13** | 'afghanistan'/de OR 'central african republic'/de OR 'libyan arab jamahiriya'/de OR 'somalia'/de OR 'south sudan'/de OR 'syrian arab republic'/de OR 'yemen'/de OR 'burkina faso'/de OR 'burundi'/de OR 'cameroon'/de OR 'congo'/de OR 'democratic republic congo'/de OR 'iraq'/de OR 'mali'/de OR 'niger'/de OR 'nigeria'/de OR 'sudan'/de OR 'chad'/de OR 'eritrea'/de OR 'gambia'/de OR 'guinea-bissau'/de OR 'haiti'/de OR 'kosovo'/de OR 'lebanon'/de OR 'myanmar'/de OR 'papua new guinea'/de OR 'venezuela'/de OR 'zimbabwe'/de OR 'comoros'/de OR 'kiribati'/de OR 'federated states of micronesia'/de OR 'timor-leste'/de OR 'tuvalu'/de OR 'melanesia'/de | 123598 | 4 May 2020 |
| **#12** | afghanistan*:ti,ab,kw OR ((central NEXT/0 african NEXT/0 republic):ti,ab,kw) OR libya*:ti,ab,kw OR somalia*:ti,ab,kw OR ((south NEXT/0 sudan*):ti,ab,kw) OR syria*:ti,ab,kw OR syrie*:ti,ab,kw OR yemen*:ti,ab,kw OR ((burkina NEXT/0 faso*):ti,ab,kw) OR burundi*:ti,ab,kw OR cameroon*:ti,ab,kw OR congo*:ti,ab,kw OR iraq*:ti,ab,kw OR irak*:ti,ab,kw OR mali*:ti,ab,kw OR niger*:ti,ab,kw OR nigeria*:ti,ab,kw OR sudan*:ti,ab,kw OR chad*:ti,ab,kw OR eritrea*:ti,ab,kw OR gambia*:ti,ab,kw OR 'guinea bissau*':ti,ab,kw OR haiti*:ti,ab,kw OR kosovo*:ti,ab,kw OR leban*:ti,ab,kw OR liban*:ti,ab,kw OR l$bnan*:ti,ab,kw OR liberia*:ti,ab,kw OR myanmar*:ti,ab,kw OR ((papua NEXT/0 new NEXT/0 guinea*):ti,ab,kw) OR venezuel*:ti,ab,kw OR zimbabw*:ti,ab,kw OR comoros:ti,ab,kw OR kiribati*:ti,ab,kw OR ((marshall NEXT/0 island*):ti,ab,kw) OR micronesia*:ti,ab,kw OR 'timor leste':ti,ab,kw OR tuvalu:ti,ab,kw OR palestin*:ti,ab,kw OR ghazza:ti,ab,kw OR ghaza:ti,ab,kw OR gaza:ti,ab,kw OR ((west* NEAR/1 bank):ti,ab,kw) OR ((solomon NEXT/0 islands):ti,ab,kw) | 1021477 | 4 May 2020 |
| **#11** | #9 OR #10 | 3187204 | 4 May 2020 |
| **#10** | research*:ti,ab,kw OR ((medicine NEAR/1 investigat*):ti,ab,kw) OR ((health*:ti,ab,kw OR retrospective:ti,ab,kw OR cohort:ti,ab,kw OR prospective:ti,ab,kw) AND (volunteer$:ti,ab,kw OR participant$:ti,ab,kw)) | 2656027 | 4 May 2020 |
| **#9** | 'research ethics'/exp OR 'dual use research'/de OR 'research subject'/exp OR 'research'/exp | 885658 | 4 May 2020 |
| **#8** | #6 OR #7 | 2817252 | 4 May 2020 |
| **#7** | (((medical OR nurs* OR dental) NEXT/1 (student$ OR graduate$ OR intern$ OR fellow$)):ti,kw) OR ((labor NEAR/3 coach*):ti,ab,kw) OR md:ti,ab,kw OR anatomist*:ti,ab,kw OR audiologist*:ti,ab,kw OR coroner$:ti,ab,kw OR (((health* OR hospital OR medical OR clinic* OR inpatient* OR outpatient*) NEAR/3 (examiner* OR assistant$ OR administrator* OR supervisor* OR receptionist* OR personnel OR team OR staff OR worker* OR researcher OR researchers OR librarian* OR informationist* OR technician* OR manager* OR housekeeper* OR janitor* OR officer* OR responder* OR paramedic* OR laboratorian* OR coordinator* OR navigator* OR attendant* OR scientist* OR cashier$ OR accountant$ OR registrar$ OR provider* OR chaperone$ OR 'man power' OR manpower OR workforce* OR caregiver$)):ti,ab,kw) OR (((er OR emergency OR '911') NEXT/1 dispatcher*):ti,ab,kw) OR epidemiologist*:ti,ab,kw OR midwi$e*:ti,ab,kw OR ((medical NEXT/1 care NEXT/1 giver$):ti,ab,kw) OR ((hospital NEXT/1 care NEXT/1 giver$):ti,ab,kw) OR ((clinical NEXT/1 care NEXT/1 giver$):ti,ab,kw) OR ((house NEXT/1 officer$):ti,ab,kw) OR resident$:ti,ab,kw OR nurse$:ti,ab,kw OR nursing:ti,ab,kw OR orderly:ti,ab,kw OR orderlies:ti,ab,kw OR dieti$ian*:ti,ab,kw OR psychologist*:ti,ab,kw OR counselor*:ti,ab,kw OR therapist*:ti,ab,kw OR pharmacist*:ti,ab,kw OR hygeinist*:ti,ab,kw OR phlebotomist*:ti,ab,kw OR chiropracter*:ti,ab,kw OR acupuncturist*:ti,ab,kw OR an$esthesiologist*:ti,ab,kw OR an$esthetist*:ti,ab,kw OR perfusionist*:ti,ab,kw OR surgeon*:ti,ab,kw OR p$ediatrician*:ti,ab,kw OR obstetrician*:ti,ab,kw OR gyn$ecologist*:ti,ab,kw OR radiologist*:ti,ab,kw OR sonographer*:ti,ab,kw OR cardiologist*:ti,ab,kw OR gastroenterologist*:ti,ab,kw OR hepatologist*:ti,ab,kw OR endocrinologist*:ti,ab,kw OR diabetologist*:ti,ab,kw OR internist*:ti,ab,kw OR hospitalist*:ti,ab,kw OR intensivist*:ti,ab,kw OR rheumatologist*:ti,ab,kw OR immunologist*:ti,ab,kw OR dermatologist*:ti,ab,kw OR allergist*:ti,ab,kw OR otolaryngologist*:ti,ab,kw OR otorhinolaryngologist*:ti,ab,kw OR oncologist*:ti,ab,kw OR h$ematologist*:ti,ab,kw OR orthop$edist*:ti,ab,kw OR physiatrist*:ti,ab,kw OR podiatrist*:ti,ab,kw OR pathologist*:ti,ab,kw OR diener*:ti,ab,kw OR cytologist*:ti,ab,kw OR cytogeneticist*:ti,ab,kw OR geneticist*:ti,ab,kw OR h$ematopathologist*:ti,ab,kw OR neurologist*:ti,ab,kw OR neurosurgeon*:ti,ab,kw OR ophthalmologist*:ti,ab,kw OR optician*:ti,ab,kw OR urologist*:ti,ab,kw OR nephrologist*:ti,ab,kw OR pulmonologist*:ti,ab,kw OR neonatologist*:ti,ab,kw OR psychiatrist*:ti,ab,kw OR physiotherapist*:ti,ab,kw OR dentist*:ti,ab,kw OR prosthodontist*:ti,ab,kw OR periodontist*:ti,ab,kw OR endodontist:ti,ab,kw OR nutritionist*:ti,ab,kw OR optometrist*:ti,ab,kw OR geriatrician*:ti,ab,kw OR gerontologist*:ti,ab,kw OR otologist*:ti,ab,kw OR feldsher*:ti,ab,kw OR exodontist*:ti,ab,kw OR doula*:ti,ab,kw | 2075089 | 4 May 2020 |
| **#6** | 'health care personnel'/exp OR 'health student'/exp | 1548681 | 4 May 2020 |
| **#5** | #1 OR #2 OR #3 OR #4 | 4335335 | 4 May 2020 |
| **#4** | 'capacity building'/de OR 'capacity building' OR 'health promotion'/exp OR 'health promotion' OR 'education'/exp OR 'education' OR 'mentoring'/exp OR 'mentoring' OR 'educational technology'/exp OR 'educational technology' OR 'webcast'/exp OR 'webcast' OR 'videoconferencing'/exp OR 'videoconferencing' OR 'learning'/exp OR 'learning' OR 'teaching'/exp OR 'teaching' OR 'distance education'/exp | 2902795 | 4 May 2020 |
| **#3** | course*:ti,ab,kw OR webinar*:ti,ab,kw OR 'e learning':ti,ab,kw OR elearning:ti,ab,kw OR learn*:ti,ab,kw OR 'online learn*':ti,ab,kw OR educat*:ti,ab,kw OR teach*:ti,ab,kw OR 'teach back':ti,ab,kw OR workshop*:ti,ab,kw OR ((promotion* NEAR/3 item*):ti,ab,kw) OR ((health NEAR/3 fair*):ti,ab,kw) OR moocs:ti,ab,kw OR train*:ti,ab,kw OR tot:ti,ab,kw OR ((pedagogic* NEAR/1 approach*):ti,ab,kw) OR webcast*:ti,ab,kw OR tutorial*:ti,ab,kw OR ((discussion NEXT/1 (forum OR board)):ti,ab,kw) OR videoconferenc*:ti,ab,kw OR 'video conferenc*':ti,ab,kw OR moodle:ti,ab,kw OR blackboard:ti,ab,kw OR (((program OR health) NEAR/3 litera*):ti,ab,kw) OR fellowship*:ti,ab,kw OR scholarship*:ti,ab,kw OR (((health OR wellness OR healthcare OR 'health care' OR smok*) NEAR/3 (promotion* OR campaign* OR awareness OR programs)):ti,ab,kw) OR (((antismok* OR 'anti smok*') NEAR/3 (promotion* OR campaign* OR awareness OR programs)):ti,ab,kw) OR ((global NEAR/2 (intervention* OR initiative*)):ti,ab,kw) OR (((healthcare OR health*) NEAR/1 (information OR promot*)):ti,ab,kw) OR ((capacity NEAR/2 building):ti,ab,kw) | 2761228 | 4 May 2020 |
| **#2** | 'access to information'/de OR 'information dissemination'/de OR 'problem solving'/de | 73531 | 4 May 2020 |
| **#1** | (((access OR freedom) NEAR/3 (open OR information)):ti,ab,kw) OR 'foia request':ti,ab,kw OR 'foia requests':ti,ab,kw OR (((information OR data) NEAR/2 (sharing* OR disseminat* OR distribut*)):ti,ab,kw) OR ((problem NEAR/3 solv*):ti,ab,kw) | 96457 | 4 May 2020 |
