# Supplementary material for: Health research capacity building of health workers in fragile and conflict-affected settings: a scoping review of challenges, strengths, and recommendations
Source: Health Res Policy Syst. 2021 May 22;19:84. doi: 10.1186/s12961-021-00725-x (PMC8140497; doi:10.1186/s12961-021-00725-x)
Supplement: Supplementary file 2 — Additional file 2: Appendix 2. List of categories, themes, codes, as well as the number of studies that included each code based on thematic analysis of studies included in the scoping review. [file 12961_2021_725_MOESM2_ESM.docx]

**Supplemental Appendix 1**

List of categories, themes, codes, as well as the number of studies that included each code based on thematic analysis of studies included in the scoping review

| **Category** | **Theme** | **Code** | **Number of studies that included code (reference)** | **Representative Quote** |
| --- | --- | --- | --- | --- |
| **Challenges to Implementing HRCB Interventions** | **Structural/ Systemic Challenges** | Partnerships | 6 (4,7,14,16,17,18) | 16. There are challenges inherent in implementing a transnational partnership such as PaM-D; in carrying out intervention research in human subjects with severe mental disorders, who are to be cared for within a newly developed partnership involving nonphysician, non-specialist actors in both formal and informal sectors of the health system and in settings prone to human right abuses; in forging partnerships among diverse stakeholders; as well as in carrying out sustainable capacity building activities among scholars and other stakeholders from diverse regions on the continent.  17. This training programme was largely driven by funding from the global North, and lacked South-South collaborations that could foster regional partnerships. |
|  |  | Local Research Context | 6 (1,4,5,6,9,12) | 1. For Atoifi researchers challenges lay in the logistics of organising the workshop and how to encourage active participation in RCS activities while continuing to operate the Hospital and College of Nursing. One Atoifi researcher demonstrated this struggle, “Nurses expressed their disappointment because they could hardly attend the research workshop - (it) is a clear indication of how they value this capacity building research workshop”.  6. In this context where research use culture is still largely undeveloped...a great deal of attention should be given to the context, the complexity, the nuances, and the interdependence of factors motivating behaviors. For example, in a country where decision making is highly centralized, any influence exerted to effect change should not be limited to the local arena, but should definitely also focus on the national level.  9. Some health workers perceived research as additional work rather than an opportunity to learn or develop professionally. |
|  |  | Sustainability | 5 (5,6,10,16,20) | 10. For the program to be sustainable, countries will ultimately have to take on the technical and logistical leadership of the program. This initiative was greatly supported by the CDC and several partners… |
|  |  | | | |
|  | **Logistical Challenges** | Human Resources | 9 (3,4,5,6,8,10,12,13,14) | 8. The high number of students dropping out or disappearing from the program has proved to be a pressing challenge... Another challenge is the lack of sufficient projects and faculty members registered in the program, which results in a number of unmatched applicants in almost every call. |
|  |  | Funding | 7 (1,9,10,13,14,17,20) | 1. There are limited funding options for health RCS in Solomon Islands and limited incentives for supporting RCS from less resourced countries. Often heath research favours commercialised, profit building technologies in resource rich countries and does not strongly support RCS in small, remote or less resourced areas such as ours in Solomon Islands |
|  |  | Duration | 7 (7,11,13,14,17,19,20) | 7. The training workshops I provided ranged in duration from 20 to 30 hours and occurred over the span of a week to a month…risk falling into a larger trend of rapid assessments, ‘helicopter ethnography’ and ‘voluntourism.’ |
|  |  | Technical Resources | 4 (1,12,17,19) | 1. The negatives for [HIC] researchers related to working in a less-resourced environment, including lack of electricity, printing, internet and other communication and health risks. "This is difficult when the internet and phone/fax are not working and there is only intermittent electricity. This makes collaborative writing of articles/ manuscripts challenging" |
|  |  | Technology | 4 (4,13,17,19) | 17. The eLearning mentoring programme was not easily adopted by the trainees. Some [] staff members did not have access to networked computers at work (e.g. the nurses and midwives), many lacked good computer literacy skills, and internet connection was erratic in Liberia. Although devices (i.e. tablets) were provided to facilitate access to the eLearning platform ... the trainees found it difficult to follow the modules and exercises. |
|  |  | | | |
|  | **Personnel Challenges** | Attitude | 6 (3,4,7,8,9,17) | 3. The challenges we noted include different levels of acceptability of the program by individual faculty and research infrastructure and lack of support by institutional leaderships. |
|  |  | Language Barriers | 5 (1,2,5,6,14) | 1. Understanding English was a challenge for some community leaders, most often during the formal presentations..As reported by one chief: When our people from the bush came down to participate, like, the Professor spoke English. When he spoke English, for those who read and write, that is good. But for us who don’t know English, we don’t understand, that is very, like, its really not good for us. |
|  |  | Learning Barriers/Academic Difficulty | 4 (1,17,18,19) | 11. All of the students said the program did not allow for enough time for the research component, with students from Haiti and Rwanda claiming that the knowledge presented was beyond their educational level. |
|  |  | | | |
|  | **Assessment & Evaluation Concerns** | Evaluation Approach | 5 (1,4,17,19,20) | 9. We were not able to include the views or observations of all members of the research teams and HCWs involved in the studies. A major omission was the collection of views and opinions of community members. We are therefore not able to fully reflect the ways by which INSPIRE may have affected on the skills and confidence of HCWs, either positively or negatively, and the community.  19. Our evaluation was limited to proximal outcomes, such as confidence and knowledge, rather than more meaningful, distal outcomes, such as publications, grants, career trajectories of participants, and change in policy or practice.  18. We were not able to compare the impacts to a control group that did not receive the capacity-building activities and so it is difficult to know what kind of changes in institutional capacity would have resulted without Emerald. |
|  |  | Evaluation Tools | 4 (17,18,19,20) | 17. ...Inadequacy of the pre- and post training test to capture the knowledge gained during the training, which included not only theoretical concepts but also skills difficult to quantify in a questionnaire. 18. It was difficult to assess practice and/or the behavioural impacts as our evaluation used proxy indicators based on self-report. |
|  |  | Participant Engagement | 2 (8,17) | 17. A moderate rate of completion may have limited the success of the training programme and the performance of the practical exercise. |
|  | | | | |
| **Strengths (Positive/Beneficial Characteristics) of HRCB Interventions** | **Locally Driven** | Context-specific Design | 7 (2,5,7,12,18,19,20) | 14. The ethical reviews for the research were discussed in length by the nursementors and mentee, including the need for each hospital engaged in the recruiting of patients to approve. By undertaking such a rigorous process, the cultural variations of the patient population were respected, laws of Nigeria governing research on persons were adhered to, and special challenges regarding scarcity of ICU medications were acknowledged.  18. Country-level adaptations were made to all of the short courses, to fit in with the individual countries’ local contexts and needs. |
|  |  | Local Collaborations | 7 (1,4,5,7,8,9,17) | 1. Numerous community leaders are now approaching Atoifi leaders requesting that their villages partner in public health research projects. This has informed a number of applications for research funds from national and international bodies.  17. The project also acknowledged the importance of engaging with the communities for the successful translation of research findings, and community members were invited to participate in the training programme. |
|  |  | Local Implementation | 5 (1,3,6,9,10) | 1. RCS undertaken at Atoifi, rather than having to leave the campus or the country, was identified as a benefit to both individual researchers and the institution. It allowed for strengthening of research knowledge and experience with a broad range of professional and community participants in the local context and using local examples. This allowed RCS while maintaining the operations of the hospital and college of nursing.  9. In all countries, project teams were involved in piloting approaches for launching, establishing, and scaling up Option B+ in the relevant health districts or states. Teams assisted with training, design of materials including site registers and monitoring tools. In Zimbabwe, INSPIRE teams also helped coordinate national stakeholder meetings, and sharing of early lessons with other implementing partners. |
|  |  | Based on Needs-Assessment | 3 (14,16,20) | 16. challenges were anticipated and carefully prepared for through the rigorously-conducted formative studies, the results of which were reflected in the protocols and were useful in the course of project implementation. |
|  |  | | | |
|  | **Pedagogical Considerations** | Practical Exercises | 7 (1, 2,5,6,11,13,17) | 1. “Benefits were also the practical outcomes that have arisen because of the workshop. . . (it has) accelerated and provided supporting evidence for a sanitation project in the village” (Australian researcher).  6. Respondents particularly appreciated the practical exercises, the discussions, and the trainers’ interactive teaching approach….They offered many examples: ‘He gave us ways of increasing membership, he prompted us to develop an action plan, and he coached us through its implementation’ (MHO respondent). |
|  |  | Interactive Learning | 6 (2,6,11,13,15,18) | 2. The sessions were interactive, with engaging discussions between the staff from the Ministry of Health and individual researchers. Participants appreciated the interactive nature of the workshop. |
|  |  | Mentorship Component | 4 (4,8,11,13) | 4. Host mentors help the trainee understand differences in local patterns of clinical care and decision making and are essential in adapting research proposals to the local context...we have found it importantn to assemble diverse mentorship resources that can offer guidance in research methodology, context expertise, and career advice. |
|  |  | | | |
|  | **Holistic CB Intervention** | Comprehensive Research Training | 4 (1,8,9,20) | 1. Taking part in all stages of the research process was identified as important. One Atoifi researcher stated, “actually taking part in the process or the stages that begins in drafting of proposal which involves consultation with the administration for consents/communities and individual…gives me the confidence to become motivated to undertake research”.  8. By providing opportunities to participate in every phase of research, the MRVP allows students to learn how to apply scientific methodology and techniques, acquire laboratory skills and improve social skills such as teamwork and oral communication, and strengthen academic credentials. |
|  |  | Multidisciplinary Population | 4 (1,10,17,18) | 10. In some countries the veterinary and laboratory sectors were included in training cohorts to foster local cross-sector collaboration and a One Health approach to surveillance and response activities |
|  |  | Mutually Beneficial | 3 (1,8,11) | 8. ..designed to serve the needs of both students and faculty members. Undergraduate students require proper exposure to research and mentorship in the medical field. On the other hand, faculty members require access to a constant supply of assistance to aid them in their research projects.  11. It became an opportunity for Canadian students to learn about research focus in developing countries.The international exchange also provided students with an opportunity to learn beyond the traditional learning environment. |
|  |  | Sustainability approach | 3 (12,13,17) | 13. To ensure sustainability after grant funding concludes, we developed a train-the-trainer workshop, which was implemented in 2014 to empower interested faculty to implement ongoing faculty development in teaching, curriculum development, and program evaluation. |
|  |  | Cost Effectiveness | 3 (1,14,19) | 14. The ethical obligations of wealthy countries to fund both nursing and medical research in resource-poor countries. The SCCM, through the Nursing Research taskforce, was able to provide a small amount of money to start the research.  19. The ToT was feasible and acceptable; additionally, the ToT was substantially lower cost per participant trained than the workshop led by an international trainer. |
|  | | | | |
| **Recommendations / Opportunities for Improvement** | **Logistics of HRCB Development and Delivery** | Training of trainers | 5 (4,8,10,18,19) | 8. Predeparture didactic sessions focused on cultural awareness, regular check-in meetings with mentors, and onsite faculty supervision are critical to deepening these skills [of cultural humility] among residents.  18. This highlights the challenges in developing training materials that could be applicable across a diverse group of countries and the importance of training local facilitators to be sensitive to the group needs when delivering and facilitating the workshops. |
|  |  | Funding | 3 (13,15,17) | 13. Interviewees reported a need for for funding to support project development. 15. Countries and many LMIC should encourage local funding towards research as a way to reduce dependency on foreign donor funding and increase research output. |
|  |  | Duration | 3 (7,8,13) | 7. The most common suggestion for improvement was to lengthen the duration of the training.  8. The extended work period will also provide students with a better chance to publish their findings. |
|  |  | Communication | 2 (14,15) | 15. Continued one-on-one mentoring at UZ and sustained communication between mentees and UB mentors is set to expand grant writing expertise and bridge the technology gap. |
|  |  | Resources | 2 (15,19) | 19. A role for HIC partner institutions may be providing database access and helping support institutional internet connectivity, |
|  |  | | | |
|  | **Structural Components of HRCB Interventions** | Evaluation Approach | 7 (1,5,6,9,17,18,19) | 9.it would have been helpful to have prospectively planned a systematic approach for evaluating capacity building activities to inform the cost effectiveness and long-term value of such research activities.  18. Considering applying triangulation techniques to assess the impact on a broader group of stakeholders and considering additional outcomes. Future evaluation frameworks should consider other ways of assessing attitudinal change and reduction in stigma, possibly using less direct proxies of this outcome. |
|  |  | Equity in Partnership Involvement | 7 (4,7,9,15,17,19,20) | 1. Another Australian researcher expressed “It would be wonderful to see ongoing exchanges of staff between Solomon Islands and Australia – both for formal training and short term exchanges”.  4. It is imperative that the high-income country partners make research capacity building and true equity in authorship as a focus.  19. more still needs to be done to ensure that LMIC researchers can access scientific knowledge on an equal footing. |
|  |  | Preparation via Needs Assessments | 3 (1,6,11) | 6. When organizing an innovation in such a context, it is important to take the time to understand the issues well and to explain the details of the action to all the stakeholders. |
|  |  | Multidisciplinary systems approach | 2 (15,16) | 15. New emphasis should be placed towards multi-dimensional systems approach, which encompasses individual training, institutional development, national health research systems and supranational health research bodies.  16. It is our recommendation that future MH capacity building activities on the continent adopt a multidisciplinary approach; and that capacity building should be linked to the overall MH research agenda of the region. |
|  |  | | | |
|  | **Content of HRCB Interventions** | Contextually relevant material | 4 (2,6,14,16) | 2. We sought to make the training relevant to the country by using some local readings and examples as often as possible...They recommended a follow-up workshop with more French material.  16. it is our recommendation that future MH capacity building... should be linked to the overall MH research agenda of the region. |
|  |  | Mentorship | 3 (10,14,15) | 10. Providing an orientation on effective mentoring techniques for staff, fully training a small group of central-level candidates in the first cohort to familiarize them with the field-based training approach and then having them serve as mentor for later cohorts, and engaging mentors from outside the country for the first few cohorts. 14. Collaboration with professional organizations to provide ongoing mentorship enriches the quality of nurse research emerging from developing countries. |
|  |  | Practical Pedagogy | 2 (2,16) | 2. They also noted some points for improvement such as: the need to develop a concrete trial proposal as part of the workshop, to reduce the amount of material covered and to allocate more time for practical sessions. 16. We also recommend that the vehicle for delivering these capacity building opportunities be a mix of formal academic degrees or fellowships as well as the regular filling of gaps in knowledge through the organising of bespoke workshops to address expressed areas of need in LMIC. |
